# Hydropriming-Mediated Metabolic Activation and Vigour Enhancement in Selected Maize (*Zea mays* L.) Landraces: Application in Ex Situ Conservation

**DOI:** 10.3390/plants15213479

**Published:** 2026-11-06

**Authors:** Marijenka Tabakovic, Vojka Babic, Vesna Peric, Vesna Dragicevic, Anika Kovincic, Violeta Oro, Rade Stanisavljevic, Ivana Zivkovic, Violeta Mickovski Stefanovic, Natalija Kravic

**Affiliations:** 1 Maize Research Institute Zemun Polje, Slobodana Bajica 1, 11185 Belgrade, Serbia; mtabakovic@mrizp.rs (M.T.); vbabic@mrizp.rs (V.B.); vperic@mrizp.rs (V.P.); vdragicevic@mrizp.rs (V.D.); anisavic@mrizp.rs (A.K.); 2 Institute for Plant Protection and Environment, Teodora Drajzera 9, 11000 Belgrade, Serbia; orovioleta65@gmail.com (V.O.); stanisavljevicrade@gmail.com (R.S.); 3 Institute for Multidisciplinary Research, University of Belgrade, Kneza Viseslava 1, 11000 Belgrade, Serbia; ivanazivkovic25@gmail.com; 4 Research and Development Institute Tamis, Novoseljanski Put 33, 26000 Pancevo, Serbia; mickovski.stefanovic@institut-tamis.rs

**Keywords:** phytate phosphorus, glutathione, total phenolics, yellow pigment, radical scavenging activity, chlorophyll synthesis, *Zea mays* L.

## Abstract

During the transition from seed quiescence to active seedling development—where early metabolic repair and structural biomass deposition are tightly regulated by genotypic identity—hydropriming efficacy exhibits a pronounced temperature- and immersion duration-dependent trade-off. This study evaluates the capacity of hydropriming to regulate this morpho-physiological and metabolic pattern in investigated maize landraces by contrasting the unprimed baseline (T0) against treatments at 20 °C (T1–T3) and 30 °C (T4–T6) across 8-h, 16-h, and 24-h immersion periods. Under optimal eustress (during the 24-h immersion at 20 °C under T3), a high germination capacity of 94% expressed by genotype L3 relies upon active phytate phosphorus (Pphy) breakdown and robust glutathione buffering to increase chlorophyll *b* up to ~96%, whereas thermal strain (induced by 8-h to 24-h exposures under T4–T6) shifts metabolic priority downward into root-zone defences at the expense of chlorophyll accumulation (which declines up to ~88%). Under thermal strain, Pphy retention triggers a massive compensatory upregulation of redox status, expanding root glutathione synthesis up to ~282% and stimulating secondary phenylpropanoid pathways to drive root yellow pigment accumulation up to ~887%, significantly reinforcing integrated radical scavenging activity to mitigate oxidative damage. Principal component analysis resolves these responses into parallel, tissue-specific functional domains: optimal growth, thermal adjustment, metabolic latency, and suppressed vigour. Ultimately, the artificial neural network modelling establishes genetic identity as the predominant driver of overall system variance, verifying that the 24-h immersion at 20 °C triggers the physiological threshold required to maintain chlorophyll homeostasis for successful autotrophic establishment, providing a targeted pre-sowing protocol for routine maize germplasm regeneration.

## 1. Introduction

The conservation and utilisation of maize genetic resources, particularly traditional landraces, are essential in modern agriculture [1]. These local varieties possess high genetic diversity and invaluable evolutionary adaptations to specific agroecosystems, serving as a crucial germplasm base for breeding high-performing, climate-resilient modern hybrids [2]. However, global climate change drives increasingly severe meteorological shifts, such as elevated monthly average temperatures and unstable precipitation [3], which directly threaten these genetic resources by disrupting seed germination [4] and subsequent seedling emergence [5]. In temperate climates, where maize sowing increasingly encounters fluctuating early-spring thermal conditions, optimal seed germination and rapid emergence are heavily constrained by soil and air temperatures, typically requiring windows of 15–20 °C and 20–25 °C, respectively [6]. Consequently, managing the duration of the germination-to-emergence period becomes vital for achieving a successful crop stand establishment and stable yield [7,8], particularly under suboptimal seedbed conditions that induce heterogeneous germination dynamics [9]. To mitigate these temperature- and hydration-dependent abiotic challenges, advanced pre-sowing priming techniques serve as a powerful tool [10,11,12]. Within this context, hydropriming under tailored thermal and temporal regimes represents a simple and cost-effective procedure that involves the partial hydration of seeds under controlled conditions, followed by a re-drying phase [13,14]. This specific treatment is highly suitable for agricultural regions characterised by high temperatures and concomitant water deficits [15,16].

As a pre-sowing technique designed to enhance germination speed and uniformity, seed priming improves these performance parameters by accelerating water uptake, improving cellular osmotic regulation, and initiating core metabolic pathways within the seed [17,18,19]. By pre-activating vital physiological processes that persist through the subsequent drying phase, this method drives rapid metabolic reactivation and intensifies antioxidant defence systems, ultimately enhancing seed performance upon sowing [20,21,22,23,24,25,26].

At the cellular level, hydropriming triggers internal adjustments crucial for germination, including respiration, protein synthesis, and enzyme activation [27], which restore mitochondrial structural integrity, thereby enhancing adenosine triphosphate (ATP) synthesis and supplying the energy necessary for early seedling development. Furthermore, the resumed metabolic activity triggered during hydropriming directly accelerates the mobilisation of nutrient reserves stored within the maize endosperm. This treatment pre-activates crucial hydrolytic enzymes, particularly α- and β-amylases, which enhance the degradation of starch into soluble sugars to efficiently fuel subsequent embryonic elongation upon sowing [24,28]. Simultaneously, hydropriming influences hormonal modulation, augments the antioxidant defence mechanism, and induces notable gene expression and epigenetic modifications, ultimately improving seed performance under both stress and non-stress conditions [29].

Among these metabolic adjustments, a highly specific biochemical hallmark of hydropriming is its regulatory influence on phytate metabolism. This process triggers the accelerated activation of phytase—an acid phosphatase enzyme that catalyses the breakdown of phytic acid, the primary storage form of phosphorus quantified as phytate phosphorus (Pphy). Depending on the species and environmental conditions, Pphy can account for up to 87% of total seed phosphorus. In the maize kernel, this reserve is concentrated predominantly within the embryo—specifically the scutellum—and, to a lesser extent, the aleurone layer [30]. The priming-induced degradation of these phytate reserves is critical; it liberates the inorganic phosphorus (Pi) required to fuel early ATP synthesis during mitochondrial aerobic respiration, while simultaneously releasing essential divalent cations necessary for rapid root system development [31]. Furthermore, hydropriming significantly enhances the seed’s antioxidant defence capacity by stimulating the non-enzymatic scavenging system—comprising reduced glutathione (GSH), phenolics, and yellow pigment (representing the lipophilic carotenoid pool)—to effectively quench reactive oxygen species (ROS). Concurrently, phytic acid (analytically quantified via its structural component, Pphy) primarily suppresses the generation of highly destructive hydroxyl radicals via transition metal chelation [32,33,34,35,36], although it can alternatively exert pro-oxidant effects depending on transition metal homeostasis. This enhanced radical scavenging capacity [37], which is typically quantified via the 2,2-diphenyl-1-picrylhydrazyl (DPPH) assay [38,39], primes the metabolic machinery to mitigate environmental stress. Consequently, this metabolic readiness preserves the photosynthetic performance of developing chloroplasts during early seedling growth [40], ensuring the timely synthesis of chlorophylls required for robust autotrophic establishment.

Such physiological revitalisation holds critical implications for the conservation of genetic resources. While modern hybrids consistently benefit from priming under stress, traditional landraces inherently exhibit high genetic and phenotypic heterogeneity in germination dynamics. Furthermore, managing extensive germplasm collections poses significant logistical challenges, as delayed regeneration often exposes accessions to physiological ageing during medium-term cold storage. Therefore, pre-sowing seed priming can serve as a complementary ex situ conservation tool to counteract viability loss and ensure successful genetic resource regeneration. To optimise this approach, selecting appropriate thermal and temporal priming regimes is essential. Within the framework of the well-established triphasic model of seed imbibition [41], hydropriming intervals must be precisely tailored to maximise Phase II (the metabolic activation phase) without triggering radicle protrusion. In this study, temperatures of 20 °C (representing a standard thermal regime for maize germination) and 30 °C serve to evaluate the potential for accelerated metabolic reactivation, while immersion durations of 8, 16, and 24 h are applied to encompass the critical temporal window of Phase II and determine the optimum duration for physiological stimulation under different thermal regimes [42].

Accordingly, this study aims to elucidate the genotype-specific responsiveness of four traditional maize landraces originating from diverse agroecological environments to these established hydropriming regimes. Particular emphasis is placed on evaluating seed germination capacity and seedling morpho-physiological parameters while comprehensively analysing the associated dynamic alterations in the contents of Pphy, GSH, total water-soluble phenolics, yellow pigment, and the chlorophyll pool, alongside the evaluation of overall radical scavenging activity. Driven by the metabolic reactivation initiated during seed imbibition, this study hypothesizes that optimising the thermal and temporal windows of hydropriming stimulates crucial seed-reserve mobilisation and biochemical adjustments, thereby establishing a pre-germinative metabolic priming that enhances the subsequent seedling transition to autotrophic growth, as verified through chlorophyll accumulation.

## 2. Results

To comprehensively evaluate the morpho-physiological and biochemical responsiveness of maize landraces to hydropriming, seed germination capacity, early seedling growth, and tissue-specific (root and shoot) biochemical profiles are systematically analysed across treatments T1–T6 relative to the unprimed control (T0). To synthesise the complex, multivariate response and elucidate genotype- and treatment-dependent inter-parameter associations, Pearson’s correlation matrices and multivariate principal component analysis (PCA) are applied to the integrated dataset. Furthermore, to capture complex non-linear interactions and establish a strict hierarchical ranking of experimental factors, an artificial neural network (ANN) model is deployed to determine relative predictor importance scores. This combined analytical approach effectively delineates key parameter-association patterns, physiological trade-offs, and factor-level predictive weights, thereby highlighting the principal variables and directional vectors driving the systemic responsiveness to temperature- and immersion interval-dependent hydropriming regimes.

### 2.1. Effects of Hydropriming on Germination Capacity, Early Seedling Growth, and Tissue-Specific Biochemical Profiles

**Figure 1:**
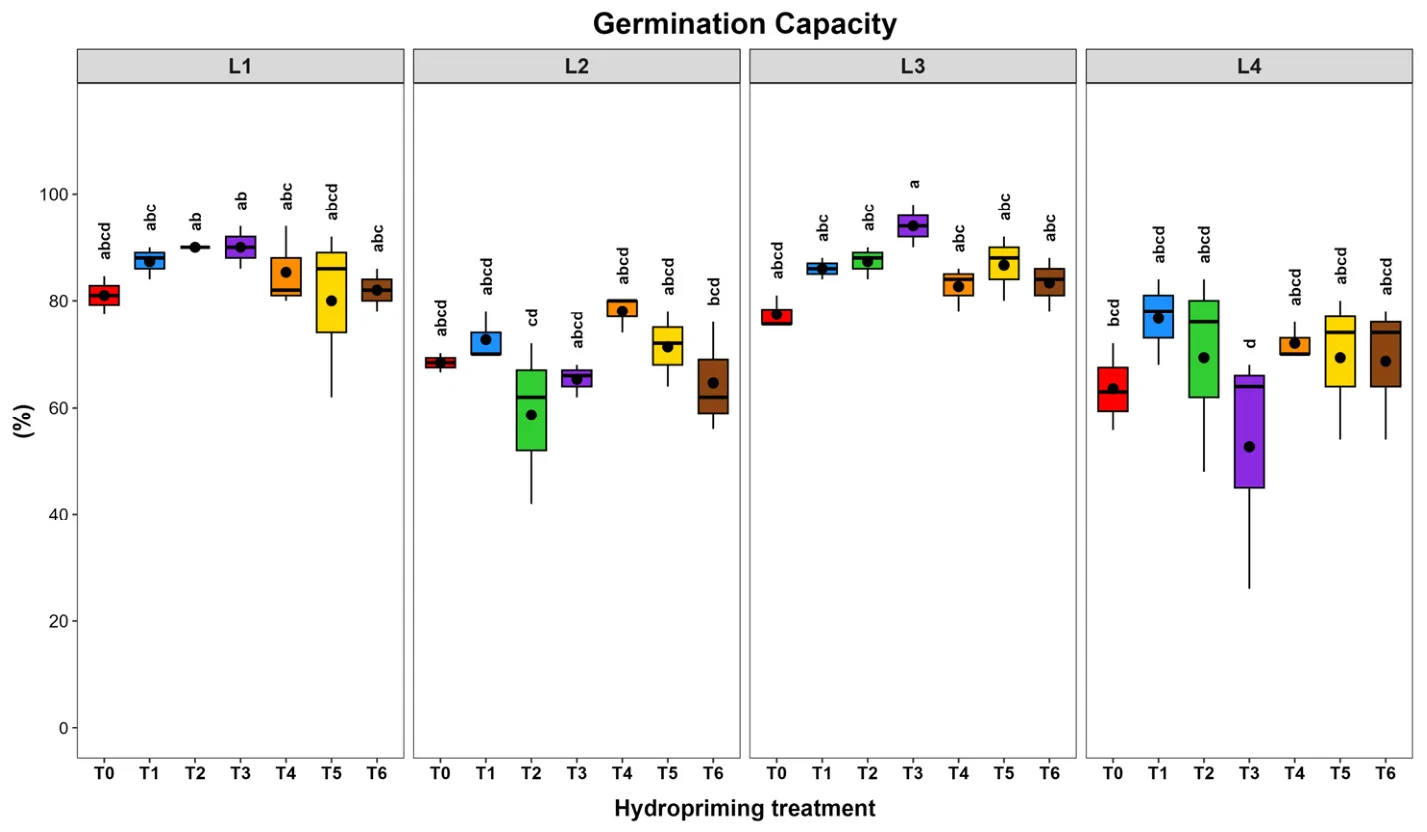
Effects of hydropriming on germination capacity across maize local landraces (L1–L4). Each boxplot represents the median and interquartile range, with whiskers extending to the minimum and maximum values of three independent biological replicates (*n* = 3). Treatments correspond to: T0 (unprimed control); T1 (20 °C, 8 h); T2 (20 °C, 16 h); T3 (20 °C, 24 h); T4 (30 °C, 8 h); T5 (30 °C, 16 h); and T6 (30 °C, 24 h). Different lowercase letters above the boxplots indicate statistically significant differences for the treatment–landrace interaction according to Tukey’s HSD test (*p* ≤ 0.05).

Short-term immersion intervals at both temperature regimes, the T1 (20 °C, 8 h) and T4 (30 °C, 8 h) treatments, induce a consistent positive response, enhancing germination capacity across all investigated genotypes relative to the unprimed control (T0), while extended immersion under the T2 (20 °C, 16 h), T5 (30 °C, 16 h), and T6 (30 °C, 24 h) treatments predominantly maintains a non-significant stimulatory or stable trend compared to the baseline profiles. Conversely, prolonged immersion under the T3 (20 °C, 24 h) treatment induces a genotype-specific response in germination capacity, eliciting a significant increase of 21.4% exclusively within genotype L3 (Figure 1, Table S1).

**Figure 2:**
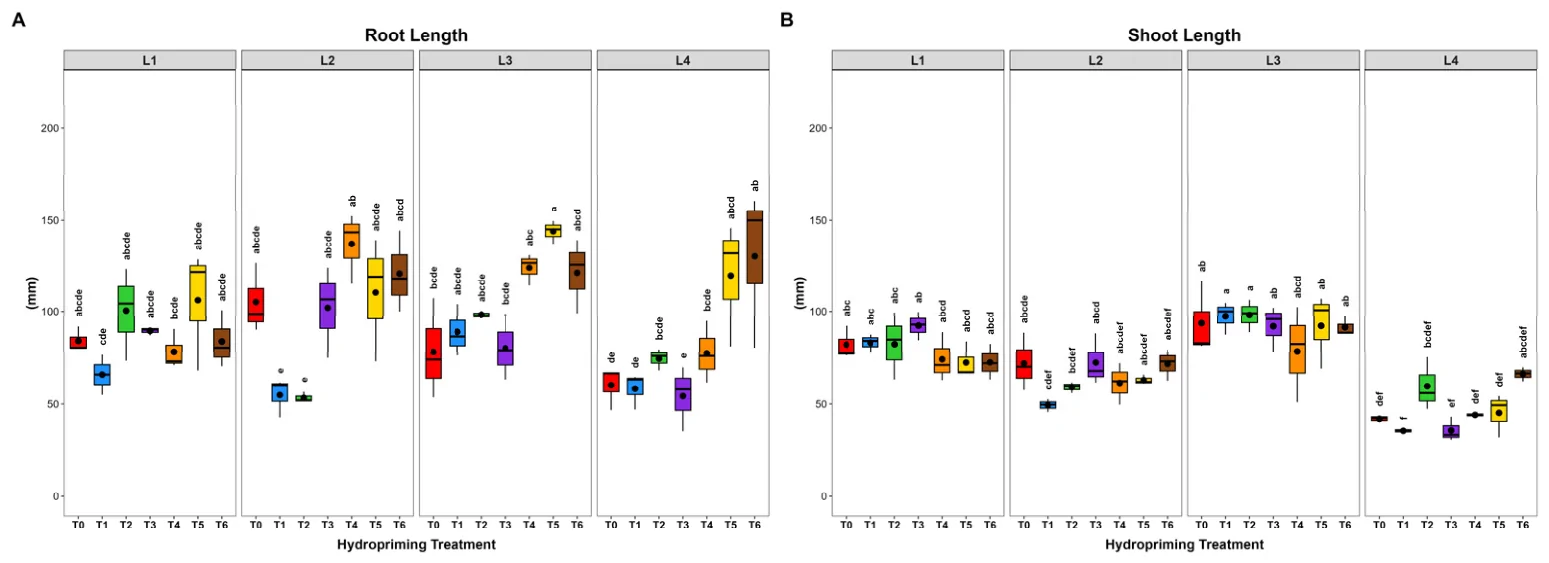
Effects of hydropriming treatments on seedling (**A**) root length and (**B**) shoot length across maize local landraces (L1–L4). Each boxplot represents the median and interquartile range, with whiskers extending to the minimum and maximum values of three independent biological replicates (*n* = 3). Treatments correspond to: T0 (unprimed control); T1 (20 °C, 8 h); T2 (20 °C, 16 h); T3 (20 °C, 24 h); T4 (30 °C, 8 h); T5 (30 °C, 16 h); and T6 (30 °C, 24 h). Different lowercase letters above the boxplots indicate statistically significant differences for the treatment–landrace interaction according to Tukey’s HSD test (*p* ≤ 0.05).

The T6 (30 °C, 24 h) and T5 (30 °C, 16 h) treatments promote the most pronounced root elongation, increasing root length by an average of 62% and 53%, respectively, followed by the T4 (30 °C, 8 h; 39%) and T2 (20 °C, 16 h; 23%) treatments. Conversely, shoot elongation is largely constrained across most landraces, with marked positive responsiveness restricted exclusively to the genotype L4 under the T2 (20 °C, 16 h; 42%) and T6 (30 °C, 24 h; 58%) treatments (Figure 2, Table S1).

**Figure 3:**
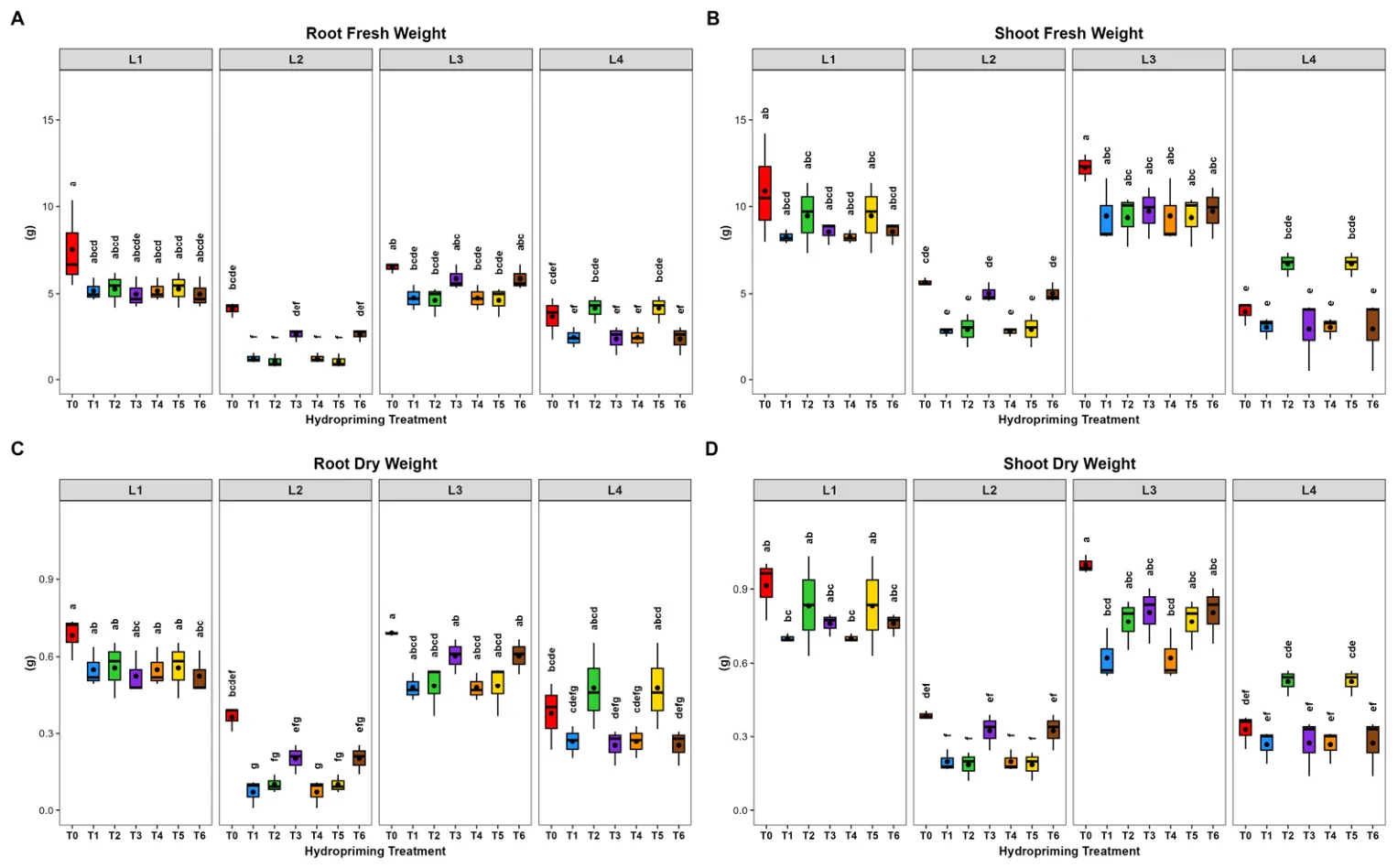
Effects of hydropriming on seedling (**A**) root fresh weight, (**B**) shoot fresh weight, (**C**) root dry weight, and (**D**) shoot dry weight across maize local landraces (L1–L4). Each boxplot represents the median and interquartile range, with whiskers extending to the minimum and maximum values of three independent biological replicates (*n* = 3). Treatments correspond to: T0 (unprimed control); T1 (20 °C, 8 h); T2 (20 °C, 16 h); T3 (20 °C, 24 h); T4 (30 °C, 8 h); T5 (30 °C, 16 h); and T6 (30 °C, 24 h). Different lowercase letters above the boxplots indicate statistically significant differences for the treatment–landrace interaction according to Tukey’s HSD test (*p* ≤ 0.05).

Overall, hydropriming predominantly exerts an inhibitory effect on both fresh and dry biomass accumulation across the majority of genotypes. Significant positive responsiveness is restricted exclusively to the genotype L4 under the T2 (20 °C, 16 h) and T5 (30 °C, 16 h) treatments, both of which elicit equivalent stimulatory effects, resulting in increases of 13% for root fresh weight (RFW), 70% for shoot fresh weight (SFW), 26% for root dry weight (RDW), and 60% for shoot dry weight (SDW), respectively (Figure 3, Table S1).

**Figure 4:**
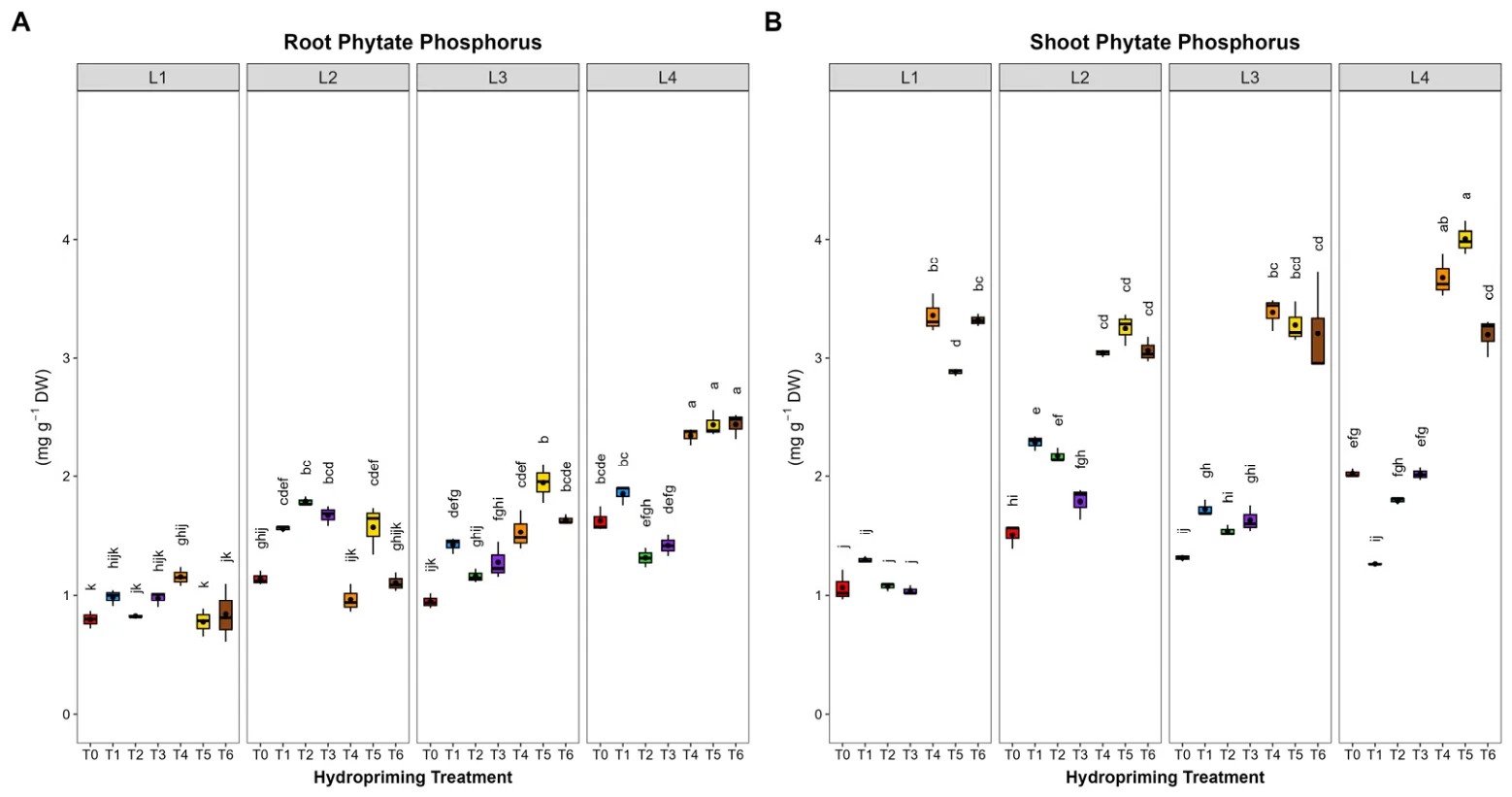
Effects of hydropriming on phytate phosphorus content in seedling (**A**) root and (**B**) shoot across maize local landraces (L1–L4). Each boxplot represents the median and interquartile range, with whiskers extending to the minimum and maximum values of three independent biological replicates (*n* = 3). Treatments correspond to: T0 (unprimed control); T1 (20 °C, 8 h); T2 (20 °C, 16 h); T3 (20 °C, 24 h); T4 (30 °C, 8 h); T5 (30 °C, 16 h); and T6 (30 °C, 24 h). Different lowercase letters above the boxplots indicate statistically significant differences for the treatment–landrace interaction according to Tukey’s HSD test (*p* ≤ 0.05).

Tissue-specific biochemical profiling reveals that hydropriming treatments applied under the elevated temperature regimes (30 °C; T4–T6) result in a substantially greater phytate phosphorus (Pphy) retention across both vegetative fractions—an accumulation trend that is more pronounced in the seedling shoot than in the root. In the seedling root, a consistent pattern of Pphy increase occurs in response to the T1 (31% on average), followed by a predominant elevation across the remaining treatments compared to the unprimed control (T0). Concurrently, shoots display a uniform trend of marked Pphy accumulation, reaching peaks of up to 215% under the T4, 170% under the T5, and 211% under the T6, while a systemic average increase occurs under the T1 (35%) and T2 (21%). Treatment T3 induces a genotype-specific response characterised by an average increase of 22% in genotypes L2 and L3, while Pphy levels remain unchanged in genotypes L1 and L4 (Figure 4, Table S1).

**Figure 5:**
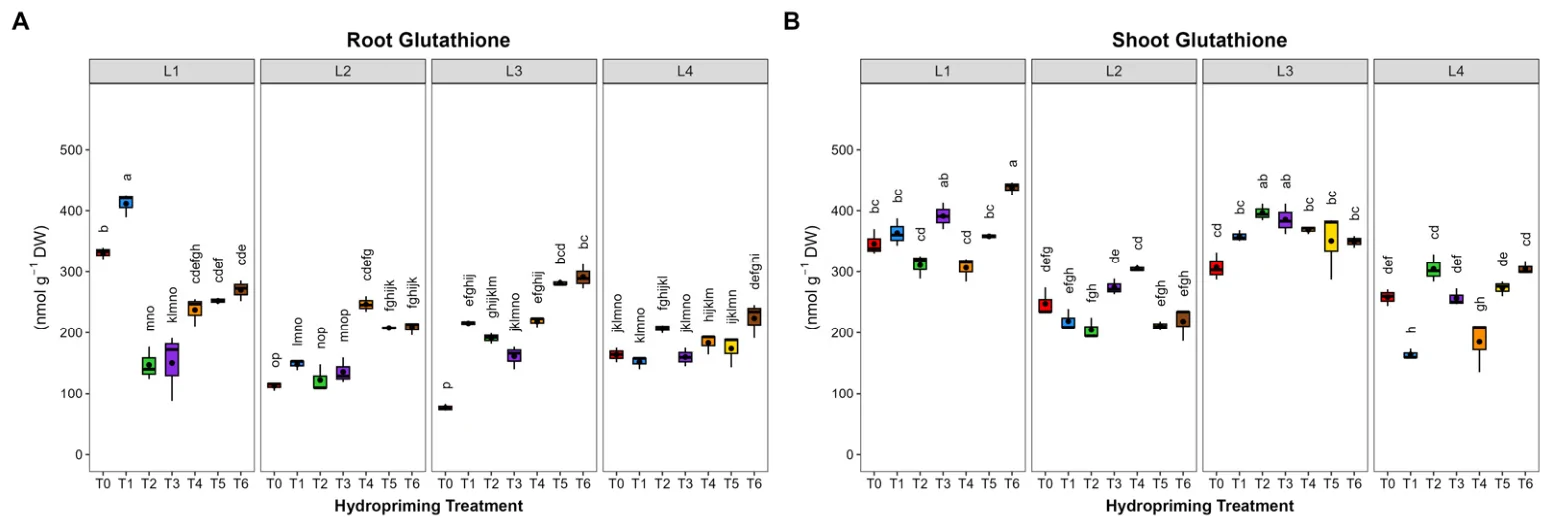
Effects of hydropriming on reduced glutathione content in seedling (**A**) root and (**B**) shoot across maize local landraces (L1–L4). Each boxplot represents the median and interquartile range, with whiskers extending to the minimum and maximum values of three independent biological replicates (*n* = 3). Treatments correspond to: T0 (unprimed control); T1 (20 °C, 8 h); T2 (20 °C, 16 h); T3 (20 °C, 24 h); T4 (30 °C, 8 h); T5 (30 °C, 16 h); and T6 (30 °C, 24 h). Different lowercase letters above the boxplots indicate statistically significant differences for the treatment–landrace interaction according to Tukey’s HSD test (*p* ≤ 0.05).

Regarding the primary redox status, the priming-induced accumulation of reduced glutathione (GSH) is substantially greater in the seedling root than in the shoot fraction. Within the root tissue, GSH accumulation is predominantly stimulated by hydropriming relative to the unprimed control (T0) in the majority of landraces, whereas prolonged hydration under the T3 induces a genotype-specific pattern. Crucially, genotype L3 exclusively displays a continuous and robust increase in root GSH content across all evaluated hydropriming treatments, reaching substantial increases that range from 111% to 282%. Concurrently, GSH accumulation within the shoot fraction is predominantly elevated under T3, T5, and T6, exhibiting a distinct upward trend across the majority of landraces, while the application of T1, T2, and T4 triggers a distinct genotype-dependent variation. Within this vegetative fraction, genotype L3 likewise maintains a steady elevation throughout all regimes, eliciting shoot GSH increases between 14% and 29% (Figure 5, Table S1).

**Figure 6:**
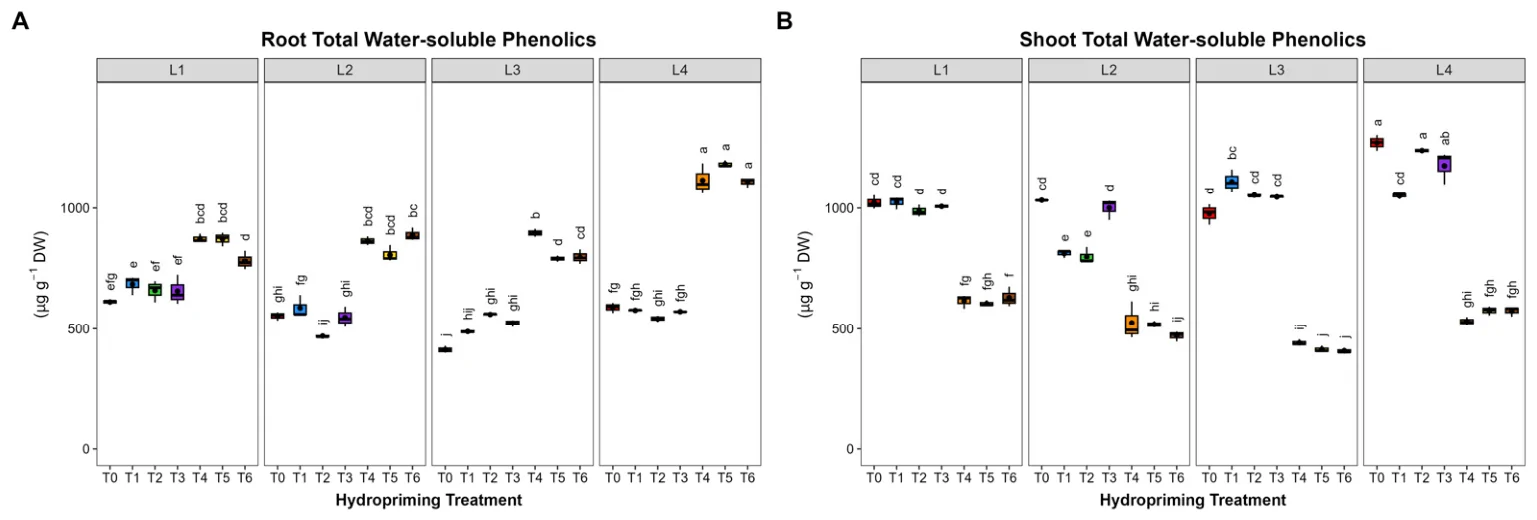
Effects of hydropriming on total water-soluble phenolic content in seedling (**A**) root and (**B**) shoot across maize local landraces (L1–L4). Each boxplot represents the median and interquartile range, with whiskers extending to the minimum and maximum values of three independent biological replicates (*n* = 3). Treatments correspond to: T0 (unprimed control); T1 (20 °C, 8 h); T2 (20 °C, 16 h); T3 (20 °C, 24 h); T4 (30 °C, 8 h); T5 (30 °C, 16 h); and T6 (30 °C, 24 h). Different lowercase letters above the boxplots indicate statistically significant differences for the treatment–landrace interaction according to Tukey’s HSD test (*p* ≤ 0.05).

With respect to the secondary metabolic profile, hydropriming under the elevated temperature acts as a powerful elicitor of phenolic synthesis in the root, while concurrently triggering a profound depletion of this antioxidant pool within the shoot fraction. Within the root tissue, this temperature-dependent response is characterised by a mild to moderate increase in total phenolic content at lower temperature regimes (T1–T3), which shifts into a highly pronounced stimulation across all landraces at the elevated temperature regimes (T4–T6), reaching substantial increases that range from 28% to 118%. Concurrently, the shoot fraction displays a contrasting pattern, characterised by minor genotype-dependent fluctuations under T1–T3, followed by a severe, systemic reduction in total phenolic levels at the elevated temperature regimes (T4–T6) across all evaluated landraces, with declines reaching up to 58% (Figure 6, Table S1).

**Figure 7:**
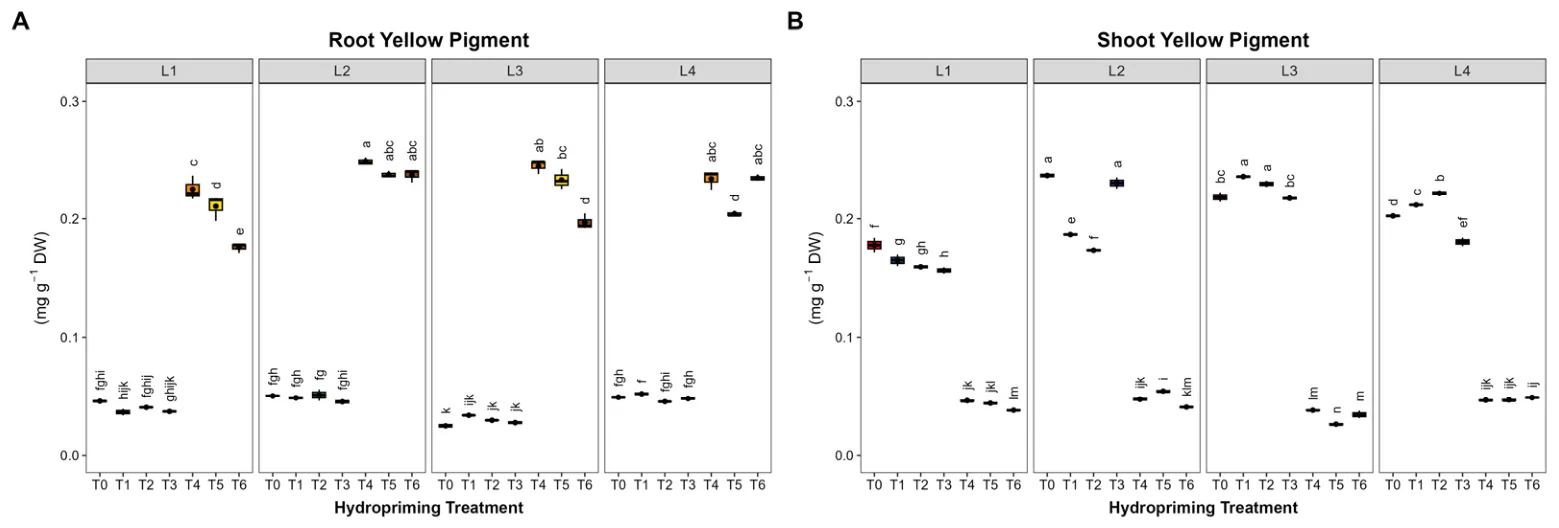
Effects of hydropriming on yellow pigment content in seedling (**A**) root and (**B**) shoot across maize local landraces (L1–L4). Each boxplot represents the median and interquartile range, with whiskers extending to the minimum and maximum values of three independent biological replicates (*n* = 3). Treatments correspond to: T0 (unprimed control); T1 (20 °C, 8 h); T2 (20 °C, 16 h); T3 (20 °C, 24 h); T4 (30 °C, 8 h); T5 (30 °C, 16 h); and T6 (30 °C, 24 h). Different lowercase letters above the boxplots indicate statistically significant differences for the treatment–landrace interaction according to Tukey’s HSD test (*p* ≤ 0.05).

The profiling of yellow pigment content reveals a highly distinct, tissue-specific response to the applied hydropriming treatments. Within the root tissue, exposure to lower temperature regimes (T1–T3) induces highly variable and localised shifts, ranging from a moderate reduction of ~21% to a moderate increase of ~36%. However, a stark metabolic shift occurs at the elevated temperature regimes (T4–T6), where a substantial surge in yellow pigment accumulation occurs across all examined landraces, with percentage increases ranging from ~283% displayed by genotype L1 under T6 to a remarkable ~887% exhibited by genotype L3 under T4. In sharp contrast, the shoot fraction exhibits a slight, stable decline at the lower temperature regimes (T1–T3), which rapidly escalates into a severe, cross-genotypic collapse of the yellow pigment pool at the elevated temperature regimes (T4–T6), driving a content drop of ~74% to ~88% (Figure 7, Table S1).

**Figure 8:**
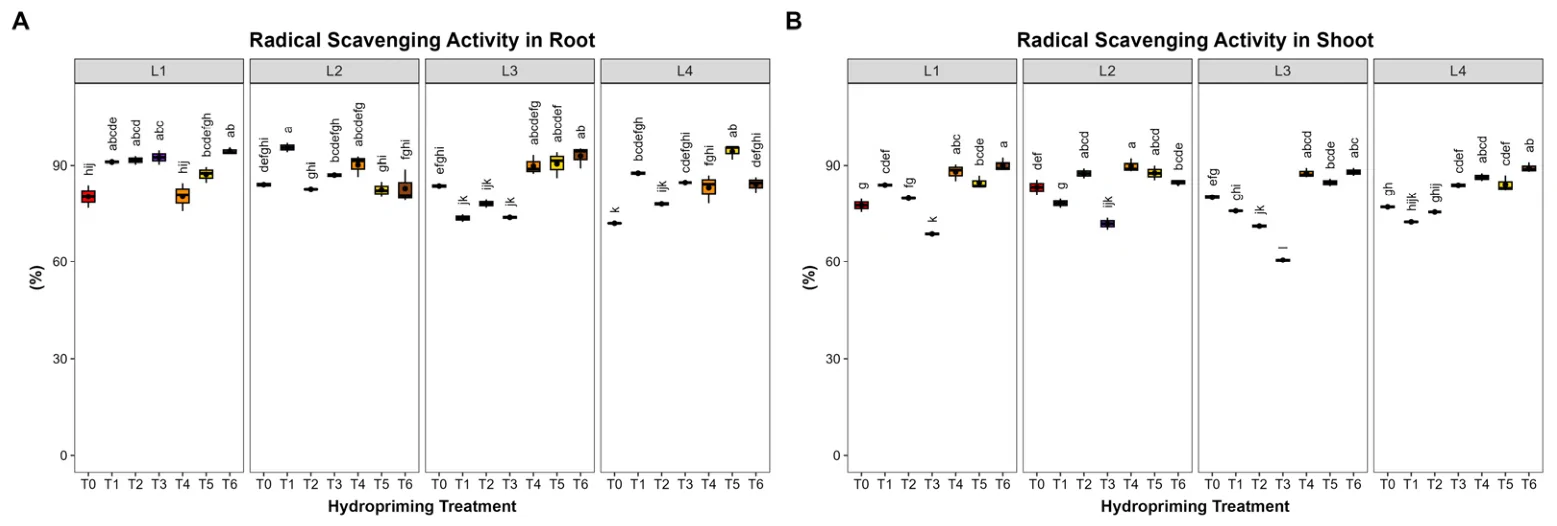
Effects of hydropriming on DPPH radical scavenging activity in seedling (**A**) root and (**B**) shoot across maize local landraces (L1–L4). Each boxplot represents the median and interquartile range, with whiskers extending to the minimum and maximum values of three independent biological replicates (*n* = 3). Treatments correspond to: T0 (unprimed control); T1 (20 °C, 8 h); T2 (20 °C, 16 h); T3 (20 °C, 24 h); T4 (30 °C, 8 h); T5 (30 °C, 16 h); and T6 (30 °C, 24 h). Different lowercase letters above the boxplots indicate statistically significant differences for the treatment–landrace interaction according to Tukey’s HSD test (*p* ≤ 0.05).

Evaluation of DPPH-based radical scavenging activity (RSA) within the seedling root demonstrates a widespread trend, with most hydropriming treatments (T1, T3, T5, and T6) eliciting a predominant, moderate increase in antioxidant capacity. In contrast, the T2 treatment reveals a highly genotype-specific response pattern across the landraces, whereas T4 prompts a uniform, slight-to-moderate increase that consistently reinforces root radical quenching capacity. Concurrently, the shoot fraction exhibits a highly coordinated, slight-to-moderate increase in antioxidant potential under the elevated temperature treatments (T4–T6), resulting in a uniform RSA increase across all investigated landraces. Conversely, lower-temperature hydropriming treatments (T1–T3) induce an inconsistent response within this vegetative fraction, characterised by widespread reductions peaking at ~25% under T3 (Figure 8, Table S1).

**Figure 9:**
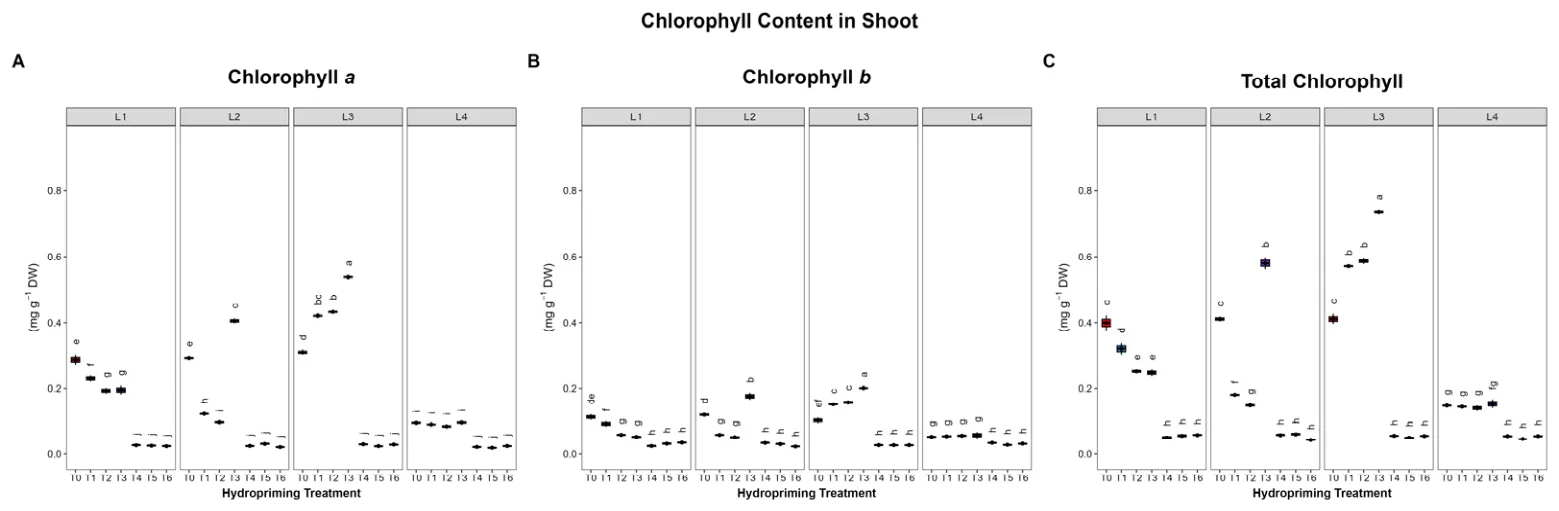
Effects of hydropriming on photosynthetic pigment contents: (**A**) chlorophyll *a*, (**B**) chlorophyll *b*, and (**C**) total chlorophyll in seedling shoots across maize local landraces (L1–L4). Each boxplot represents the median and interquartile range, with whiskers extending to the minimum and maximum values of three independent biological replicates (*n* = 3). Treatments correspond to: T0 (unprimed control); T1 (20 °C, 8 h); T2 (20 °C, 16 h); T3 (20 °C, 24 h); T4 (30 °C, 8 h); T5 (30 °C, 16 h); and T6 (30 °C, 24 h). Different lowercase letters above the boxplots indicate statistically significant differences for the treatment–landrace interaction according to Tukey’s HSD test (*p* ≤ 0.05).

Hydropriming treatments exert a pronounced, temperature-dependent influence on the photosynthetic pigment profile (chlorophyll *a*, chlorophyll *b*, and total chlorophyll) within seedling shoots. Across this metabolic pool, hydropriming under the elevated temperatures (T4–T6) causes severe and consistent reductions across all evaluated landraces compared to the unprimed control (T0). Averaged across the germplasm, treatments T4–T6 induce sharp declines of ~88% for chlorophyll *a*, ~66% for chlorophyll *b*, and ~81% for total chlorophyll content, respectively. Conversely, hydropriming at lower temperature regimes (T1–T3) elicits marked genotype-specific variations. Genotype L3 displays a consistent and prominent accumulation, whereas L1 exhibits a continuous decline in all photosynthetic fractions across these treatments. Furthermore, among these lower-temperature treatments, T3 exerts the most pronounced positive effect, promoting peak pigment accumulation across the majority of evaluated landraces. Under T3, peak increases in genotype L3 reach ~74%, ~96%, and ~79% for chlorophyll *a*, *b*, and total chlorophyll, respectively, while L2 displays corresponding increases of ~39%, ~47%, and ~41%. In contrast, genotype L4 shows no statistically significant changes under T3 (Figure 9, Table S1).

### 2.2. Treatment-Driven Alterations in Parameter Coordination and Multivariate Profiling

#### 2.2.1. Internal Correlation and Cross-Network Coordination of Morpho-Physiological and Biochemical Parameters

**Figure 10:**
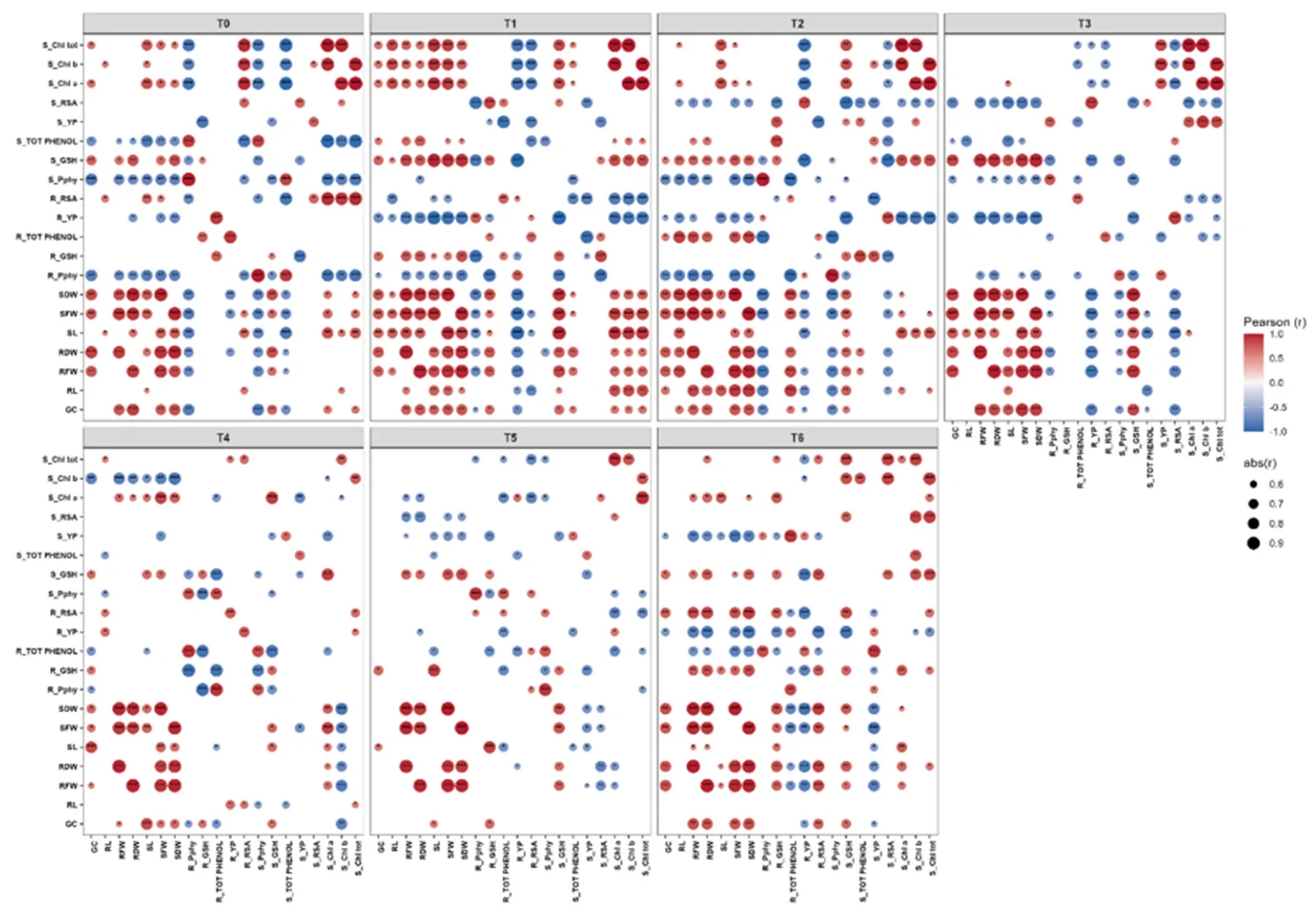
Pearson correlation matrix of morphological and biochemical parameters in maize local landraces. The correlogram illustrates the strength and direction of associations between parameters across hydropriming treatments: T0 (unprimed control); T1 (20 °C, 8 h); T2 (20 °C, 16 h); T3 (20 °C, 24 h); T4 (30 °C, 8 h); T5 (30 °C, 16 h); and T6 (30 °C, 24 h). The size and colour of the circles indicate the magnitude and direction of the Pearson correlation coefficient (r), respectively. Significance levels are indicated as follows: *—*p* < 0.05 (|r| ≥ 0.576), **—*p* < 0.01 (|r| ≥ 0.708), and ***—*p* < 0.001 (|r| ≥ 0.823) based on two-tailed critical values for a sample size of *n* = 12. Non-significant correlations are displayed as empty spaces. Abbreviations: GC—germination capacity; RL—root length; RFW—root fresh weight; RDW—root dry weight; SL—shoot length; SFW—shoot fresh weight; SDW—shoot dry weight; Pphy—phytate phosphorus; GSH—reduced glutathione; TOT PHENOL—total phenolics; YP—yellow pigment; RSA—radical scavenging activity; Chl—chlorophyll; R_—root vegetative fraction; S_—shoot vegetative fraction.

Correlation analysis delineates distinct bivariate associations within and across the evaluated morpho-physiological and biochemical parameter networks (Figure 10).

Hydropriming differentially regulates the alignment of germination capacity (GC) with seedling elongation and biomass accumulation. Notably, T3 maximises the coordination of GC with seedling biomass (r = 0.863 for RFW; r = 0.918 for SFW; r = 0.881 for SDW), whereas T4 and T2 selectively enhance GC correlations with shoot length (SL; r = 0.832) and root length (RL; r = 0.647), respectively; these associations collapse under T5, thereby eliminating the significance of GC–biomass correlations. The coordination between root and shoot parameters governing elongation and biomass accumulation further diverges across regimes. Specifically, RL shifts from an uncorrelated baseline state (T0) to peak biomass reliance under T2 (r = 0.840 for RFW; r = 0.896 for SDW) before aligning primarily with SL under T3 (r = 0.663), while SL maintains a robust coordination with SFW under T1 and T3 (r = 0.934 and r = 0.855, respectively). Conversely, exposure to elevated temperature regimes across all immersion intervals (T4–T6) induces a complete shift towards weaker or inverse correlations. Despite these variations, the investigated hydropriming regimes maintain strong and uniform positive correlations between fresh and dry weights within seedling vegetative fractions (r ≥ 0.879 for RFW vs. RDW; r ≥ 0.957 for SFW vs. SDW) and between root and shoot tissues (r ≥ 0.899 for RFW vs. SFW; r ≥ 0.895 for RDW vs. SDW), confirming that tissue biomass accumulation and partitioning remain strictly conserved across all experimental conditions (Figure 10).

Hydropriming likewise modulates the coordination between seedling elongation and tissue metabolic markers across treatments. Under unprimed T0 conditions, baseline elongation exhibits a positive reliance on constitutive root radical scavenging activity (R_RSA) (r = 0.621 for RL; r = 0.720 for SL vs. R_RSA) and shoot photosynthetic pigments (r ≥ 0.565). Hydropriming under the T1 treatment strengthens the coordination with the photosynthetic apparatus, inducing peak positive correlations between elongation and shoot chlorophylls for both RL (r ≥ 0.809) and SL (r ≥ 0.902), alongside a maximum stimulatory alignment with shoot glutathione for SL (r = 0.956). In the T2 treatment, SL maintains a strong positive correlation with photosynthetic pigments (r ≥ 0.738), whereas RL shifts towards a primary reliance on root total phenolics (r = 0.864) and exhibits an inverse correlation with phytate reserves (r = −0.813 with R_Pphy). Under the T3 and T4 treatments, positive metabolic drivers become constrained. SL retains a positive correlation with shoot glutathione in the T3 treatment (r = 0.798) but displays a distinct inverse correlation with shoot radical scavenging activity (S_RSA) and total phenolics (r ≤ −0.819). In the T4 treatment, RL exhibits a renewed positive dependence on root yellow pigment (r = 0.678) and R_RSA (r = 0.634), whereas SL displays inverse correlations with Chl *b* (r = −0.667) and root phenolics (r = −0.597). Finally, under medium-term and extended immersion intervals at elevated temperature (T5–T6), RL exhibits a total loss of metabolic responsiveness across all evaluated parameters (|r| < 0.576); conversely, SL bypasses this physiological decline by redirecting its positive dependency onto the root glutathione pool (r = 0.875 in the T5 treatment; r = 0.641 in the T6 treatment) and re-establishing a positive alignment with Chl a (r = 0.752 in the T6 treatment).

Root biomass parameters exhibit distinct correlation profiles with tissue-specific biochemical markers across hydropriming regimes. Under unprimed T0 conditions, both RFW and RDW display significant inverse correlations with Pphy in root (r = −0.736 for RFW; r = −0.721 for RDW vs. R_Pphy) and shoot (r = −0.755 for RFW; r = −0.718 for RDW vs. S_Pphy) tissues, while demonstrating a positive reliance upon shoot reduced glutathione (S_GSH; r = 0.661 for RFW; r = 0.794 for RDW). Hydropriming under lower temperature regimes (T1–T3) intensifies metabolic coordination; the T1 treatment induces a peak alignment between root mass and the GSH pool across both tissues (r ≥ 0.758 for R_GSH; r ≥ 0.763 for S_GSH), shoot total phenolics (r ≥ 0.672), and shoot photosynthetic pigments (r ≥ 0.650). In the T2 treatment, root biomass accumulation shifts towards a strong reliance on root total phenolics (r = 0.825 for RFW; r = 0.746 for RDW) alongside a persistent inverse alignment with phytate reserves (r ≤ −0.833 vs. R_Pphy). Under the T3 treatment, root weight parameters achieve their maximum positive correlation with the S_GSH pool (r = 0.924 for RFW; r = 0.920 for RDW), while maintaining an inverse relationship with root yellow pigment (r = −0.895 for RFW; r = −0.879 for RDW). Exposure to T4–T6 disrupts phytate-dependent root biomass regulation and weakens the coordination with S_GSH. Specifically, the T5 treatment induces prominent inverse correlations of root weights with S_RSA (r = −0.719 for RFW; r = −0.783 for RDW) and shoot Chl *a* (r = −0.616 for RFW; r = −0.666 for RDW), while the T6 treatment elicits a marked negative association with S_YP (r = −0.810 for RFW; r = −0.720 for RDW). Nevertheless, the T6 treatment triggers a distinct secondary metabolic recovery, wherein both root weight parameters re-establish significant positive alignments with R_GSH (r ≥ 0.735), S_GSH (r ≥ 0.663), and Chl *a* (r ≥ 0.634).

Shoot biomass parameters (SFW and SDW) also exhibit distinct correlation profiles with tissue-specific biochemical markers across regimes (Figure 10). Under unprimed T0 conditions, shoot biomass displays a strong inverse alignment with phytate reserves in both root (r ≤ −0.796) and shoot (r ≤ −0.777) tissues, alongside shoot total phenolics (r ≤ −0.699), while relying significantly upon S_GSH (r ≥ 0.682) and Chl *a* (r ≥ 0.647). Hydropriming under lower temperature regimes (T1–T3) radically reshapes this metabolic dependency; the T1 treatment induces peak positive correlations between shoot biomass and all photosynthetic pigments (r ≥ 0.725) alongside shoot total phenolics (r ≥ 0.581), whereas the T2 treatment shifts this reliance towards root total phenolics (r ≥ 0.806) under persistent phytate suppression. Under the T3 treatment, shoot biomass accumulation achieves its maximum positive correlation with the S_GSH pool (r = 0.915 for SFW; r = 0.961 for SDW), while establishing intense inverse associations against S_RSA (r ≤ −0.795) and root yellow pigment (r ≤ −0.885). Concurrently, exposure to the T4–T6 treatments predominantly induces a pronounced metabolic uncoupling; under T4, phytate-dependent regulation completely collapses, triggering a sharp inversion against Chl *b* (r ≤ −0.804) despite maintaining a positive alignment with Chl *a* (r ≥ 0.727). Under T5, shoot biomass exhibits a widespread loss of metabolic coordination across the majority of parameters, except for a persistent strong reliance upon shoot reduced glutathione (r ≥ 0.790). Finally, T6 induces a systemic realignment wherein shoot biomass parameters re-establish significant positive correlations with root and shoot glutathione pools (r ≥ 0.589) alongside the alignment of SFW with R_RSA (r = 0.800), even while displaying intense inverse alignments with root total phenolics (r ≤ −0.759) and yellow pigment extracted from both seedling vegetative fractions (r ≤ −0.808) (Figure 10).

To evaluate the treatment-dependent restructuring of root and shoot metabolic networks, correlation patterns across the T1–T6 treatments are compared against the baseline control (T0). Overall, hydropriming induces quantitative realignments and sign inversions across nutrient reserve mobilisation (Pphy), primary cellular redox status (GSH), secondary metabolites (total phenolics and yellow pigment), and radical scavenging activity (RSA), ultimately modulating photosynthetic apparatus development (Figure 10).

Under the unprimed baseline condition (T0), nutrient mobilisation displays tight inter-tissue coordination, evidenced by a near-perfect positive physiological association between root and shoot Pphy (r = 0.983). Both phytate pools align strongly with shoot total phenolics (r ≥ 0.862) whereas they exert a systemic suppression on tissue antioxidant capacity, S_GSH, and the photosynthetic pigment complex (r ≤ −0.675), validating an active physiological stimulus driven by efficient phytate mobilisation and hydrolytic reserve breakdown. Within the root tissue, R_GSH correlates positively with root total phenolics (r = 0.737) and S_GSH (r = 0.597), but inversely with shoot yellow pigment (r = −0.870). Concurrently, R_RSA operates as the primary positive driver of the photosynthetic apparatus, exhibiting exceptionally strong positive alignments with the chlorophyll pool (r ≥ 0.907) and S_RSA (r = 0.703). Conversely, shoot total phenolics exhibit intense inverse associations with chlorophyll accumulation (r ≤ −0.865), while photosynthetic pigments maintain stringent internal collinearity (r ≥ 0.944).

Under T1–T3, the network undergoes internal realignment towards the glutathione pool and photosynthetic pigments. In T1, the baseline inverse reliance on Pphy shifts into localised negative alignments against R_GSH (r = −0.911) and S_RSA (r = −0.910), while R_GSH correlates positively with root total phenolics, S_GSH, and S_RSA (r = 0.637, r = 0.733, and r = 0.845, respectively), enabling S_GSH and shoot total phenolics to drive photosynthetic pigments (r ≥ 0.798 and r ≥ 0.576). Under T2, root Pphy shifts into an intense inverse correlation with root total phenolics (r = −0.935) and S_GSH (r = −0.663); concurrently, R_GSH correlates strongly with S_GSH (r = 0.688) and shoot total phenolics (r = 0.863), while S_GSH drives the chlorophyll pool (r ≥ 0.788) but inversely aligns with S_RSA (r = −0.940). This coordination culminates under T3, where inter-tissue Pphy coordination relaxes (r = 0.770), and root and shoot Pphy display inverse relationships with S_GSH (r = −0.770 and r = −0.798, respectively) alongside R_Pphy vs. R_total phenolics (r = −0.619). Concurrently, a prominent sign inversion occurs under T3, where shoot yellow pigment aligns positively with the chlorophyll pool (r ≥ 0.782), whereas root total phenolics (r ≤ −0.575) and S_RSA (r ≤ −0.647) display intense inverse associations with the developing photosynthetic apparatus, while photosynthetic pigments preserve rigid internal collinearity (r ≥ 0.960).

Under T4–T6, the network undergoes distinct sign inversions, inducing a pronounced metabolic uncoupling. Under T4 and T5, the baseline inverse character of phytate completely collapses; root Pphy establishes strong positive correlations with root total phenolics (r = 0.920 in T4) and R_RSA (r = 0.599 in T5) while exhibiting an extensive inverse correlation with total chlorophyll under T5 (r = −0.614), reflecting severe tissue-level stress. In T4, this transition is preceded by a temporary disruption in photosynthetic collinearity via a Chl *a* vs. Chl *b* inversion (r = −0.577), alongside a sharp inversion against Chl *b* (r ≤ −0.804) despite maintaining a positive alignment with Chl *a* (r ≥ 0.727). Simultaneously, under T5, shoot biomass exhibits a widespread loss of metabolic coordination across the majority of markers, except for a persistent strong reliance upon shoot reduced glutathione (r ≥ 0.790), while R_GSH displays intense inverse relationships with root total phenolics (r = −0.729) and shoot yellow pigment (r = −0.738), while R_RSA correlates negatively with Chl a (r = −0.796) and total chlorophyll (r = −0.750), leaving S_GSH as a strong anchor that correlates negatively with shoot yellow pigment (r = −0.705). Finally, T6 triggers a compensatory secondary metabolic realignment. Within the root tissues, R_GSH re-establishes dominant positive control, aligning strongly with R_RSA (r = 0.758), S_GSH (r = 0.706), and Chl *a* (r = 0.764), whereas root total phenolics inversely align with R_RSA (r = −0.627) and R_YP displays intense negative alignments with R_RSA (r = −0.867) and S_GSH (r = −0.886). Concurrently, shoot S_GSH and S_RSA form a coordinated positive defence barrier, achieving robust correlations with Chl *b* (r = 0.805 and r = 0.858) and total chlorophyll (r = 0.844 and r = 0.873), while shoot biomass parameters re-establish strong positive correlations with root and shoot glutathione pools (r ≥ 0.589) alongside SFW and R_RSA (r = 0.800). This realignment occurs even while shoot total phenolics correlate positively with Chl *b* (r = 0.790), R_RSA inversely aligns with shoot yellow pigment (r = −0.624), and photosynthetic markers display intense inverse alignments with root yellow pigment (r ≤ −0.584 for Chl *b*; r ≤ −0.686 for total chlorophyll) (Figure 10).

#### 2.2.2. Multivariate Profiling and Hydropriming-Driven Separation of Target Parameters

To complement bivariate correlations and identify the primary drivers of variability, an integrated Principal Component Analysis (PCA) resolves the multivariate network coordination of morpho-physiological and biochemical variables governing hydropriming responses across the evaluated maize landraces. This multivariate synthesis yields two principal components (PC1 and PC2) with eigenvalues greater than 1.0 for both seedling root and shoot datasets, which cumulatively account for 65.92% and 83.00% of the total tissue-specific morpho-physiological and biochemical variance, respectively (Table 1).

**Figure 11:**
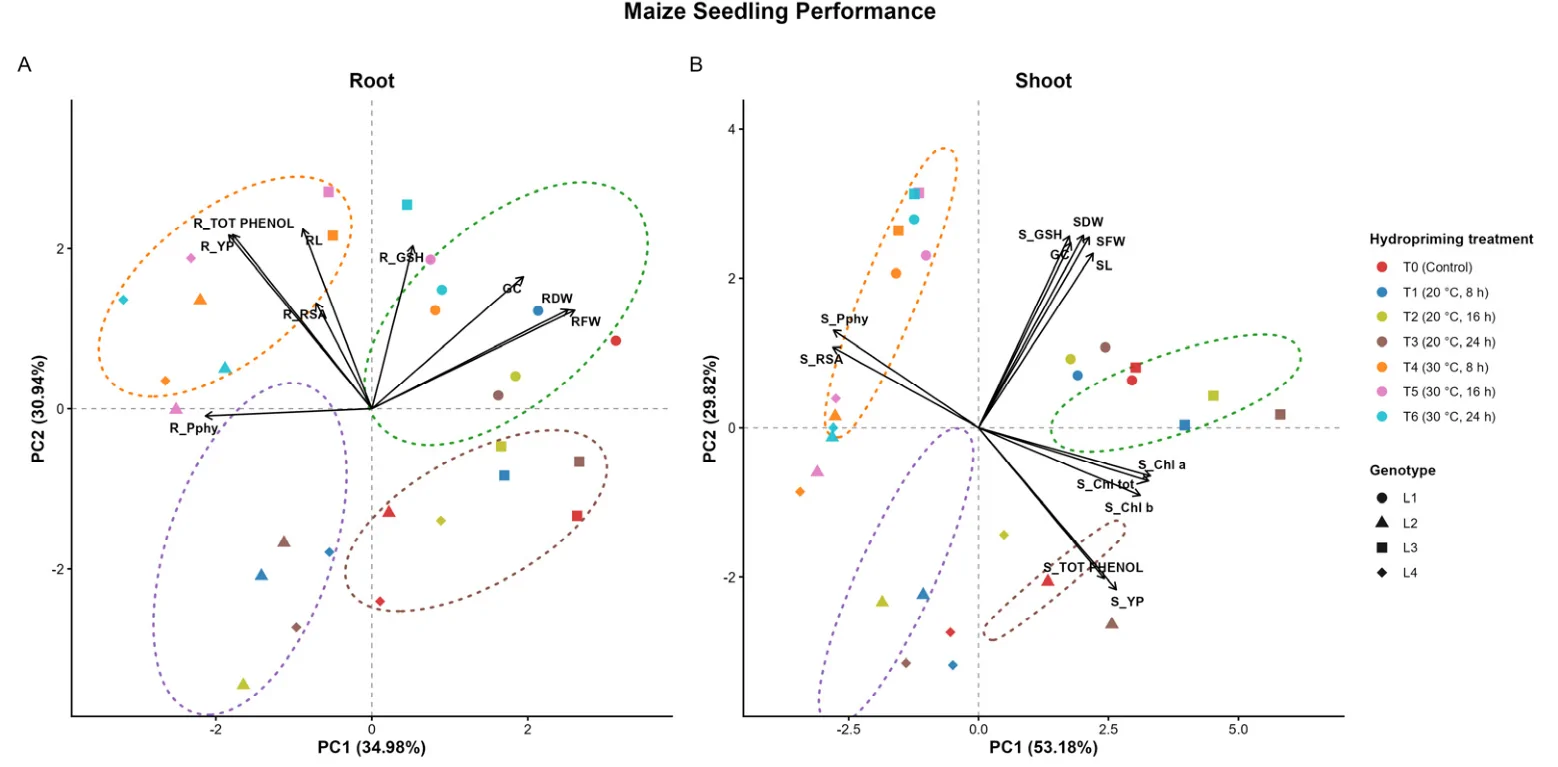
PCA biplot illustrating the correlation between morpho-physiological and biochemical parameters in seedling (**A**) root and (**B**) shoot tissues across unprimed and hydroprimed maize landraces. Each individual plot symbol represents the mean value of a specific Genotype × Treatment (G × T) interaction; vectors indicate factor loadings for the integrated morpho-physiological and biochemical variables; colour coding distinguishes hydropriming treatments (Treatments T0–T6); dashed ellipses delineate 95% confidence clustering patterns across the G × T interactions. Abbreviations: Prefixes R_ and S_ denote root and shoot tissue parameters, respectively. GC–germination capacity; RL/SL–root/shoot length; FW–fresh weight; DW–dry weight; Pphy–phytic phosphorus; GSH–reduced glutathione; tot phenol–total phenolics; YP–yellow pigment; RSA–radical scavenging activity; Chl–chlorophyll (*a*, *b*, tot).

The resulting PCA plots delineate integral multivariate patterns, revealing both treatment-dependent clustering and landrace-specific metabolic divergence across the assessed morpho-physiological and biochemical markers of root and shoot tissues (Figure 11).

In the seedling root tissue, PC1 represents the primary axis of variation, accounting for 34.98% of the total variance (eigenvalue 3.15) (Table 1A and Table S2A). This axis delineates an inverse correlation between structural biomass accumulation and Pphy retention. Specifically, PC1 exhibits strong positive co-alignments for RFW (0.849) and RDW (0.822), in sharp contrast to a strong negative correlation with R_Pphy (−0.700). PC2 accounts for an additional 30.94% of the root variance (eigenvalue 2.76) and captures a coupled metabolic cascade linking early root elongation to non-enzymatic antioxidant synthesis, defined by strong positive co-alignments among RL (0.732), R_tot phenol (0.710), and R_YP (0.708).

In the seedling shoot tissue, PC1 accounts for 53.18% of the overall variance (eigenvalue 6.38) and reflects a tightly coupled coordination between photosynthetic pigment accumulation and cellular metabolic reallocation during shoot development (Table 1B and Table S2B). Highly pronounced positive co-alignments exceeding the selection threshold occur for S_Chl *a* (0.937), S_Chltot (0.927), S_Chl *b* (0.881), and S_YP (0.750). In sharp contrast, S_RSA (−0.794) and S_Pphy (−0.791) exert strong inverse correlations along PC1, underscoring a prominent tissue-specific metabolic shift. Meanwhile, PC2 accounts for 29.82% of the shoot variance (eigenvalue 3.58) and governs structural seedling vigour and redox homeostasis, being strictly defined by the tightly coordinated alignment of SDW (0.728), S_GSH (0.725), SFW (0.722), and GC (0.700).

The spatial alignment of root functional parameters across the PC1 vs. PC2 coordinate space (Figure 11A) reveals a clear gradient of physiological responses driven by hydropriming efficacy, structured into four metabolically distinct domains. The upper-right quadrant defines the optimal vigour and biomass accumulation domain, characteristically driven by co-orientated, elongated vectors for GC, RFW, RDW, and robust redox buffering via reduced glutathione (R_GSH). Genotype L1 demonstrates absolute physiological plasticity in response to hydropriming, maintaining heightened metabolic reactivity and structural vigour across all temperature regimes and immersion intervals (1.0–1.6). Genotype L3 converges upon this high-vigour trajectory exclusively under the most intensive regime (3.6), indicating that extended immersion at the elevated temperature (30 °C, 24 h) is required to overcome metabolic latency and drive maximal biomass deposition in this plane.

The upper-left quadrant functions as the root elongation and secondary metabolic defence domain, governed by maximal-length vectors for RL, R_RSA, R_tot phenol, and R_YP. This vector alignment reflects a coordinated physiological transition towards cellular elongation (RL) coupled with an enhanced secondary metabolic and antioxidant defence response (R_tot phenol, R_YP, and R_RSA) induced by hydropriming under the elevated temperature (30 °C). Ordered along this response gradient, this protective trajectory is most prominently activated in genotype L4 (4.5, 4.6, and 4.4), which exhibits the greatest magnitude of metabolic shift along the negative PC1 axis, extending markedly farther leftward along the same horizontal plane than the configuration observable in genotype L2 (2.6 and 2.4). Concurrently, the spatial displacement exhibited by genotype L3 (3.5 and 3.4) anchors the apex of this domain, displaying the strongest R_RSA response and aligning directly with maximum physiological root elongation responses.

The lower-right quadrant represents the quiescent baseline and metabolic decoupling domain. Along a decreasing physiological response gradient, this domain transitions from the moderate physiological responses of genotype L3 (3.3, followed by 3.2 and 3.1) and genotype L4 (4.2) under lower-temperature regimes towards the strictly unprimed controls shown by genotypes L3, L2, and L4 (3.0, 2.0, and 4.0). The spatial displacement of these interactions away from the primary biomass and redox vector cluster indicates a physiological decoupling of early germination events from active root structural development. Lower temperature regimes (20 °C) preserve basic structural attributes, thereby failing to impart a sufficient metabolic stimulus to trigger enhanced tissue expansion or antioxidant system activation.

The lower-left quadrant captures the phytate phosphorus retention and metabolic latency domain, dominated by the horizontal vector for R_Pphy along the negative PC1 axis. Alignment along this trajectory denotes unmobilised phytate phosphorus fractions resulting from minimal phytate cleavage. Ordered along this response gradient by decreasing magnitude of reserve retention, this latent state is most prominently expressed by genotype L2 under a medium immersion duration at the elevated temperature regime (2.5), which occupies the transition boundary along this margin. This is followed by the responses of genotype L2 (2.3, 2.2, and 2.1) and genotype L4 (4.3 and 4.1) under lower temperature regimes, which cluster deeper within this domain, demonstrating a state of delayed reserve mobilisation under non-stimulating priming conditions.

The spatial distribution of shoot physiological parameters across the PC1 vs. PC2 coordinate space (Figure 11B) captures the shoot-specific metabolic and structural reconfiguration driven by hydropriming, partitioning performance into four distinct metabolic domains.

The upper-right quadrant defines the optimal growth, vigour, and biomass accumulation domain, strongly governed by co-oriented, elongated vectors for SL, SFW, SDW, GC, and S_GSH. This domain consolidates the highly responsive developmental trajectories of genotype L1 and genotype L3 under unprimed baseline and lower-temperature regimes, spanning treatments 1.0–1.3 and 3.0–3.3 along the biomass accumulation gradient. These spatial positions demonstrate that both genotype 1 and genotype 3 possess high intrinsic shoot vigour, allowing both the inherent baseline potential and lower-temperature priming regimes (20 °C for 8 h, 16 h, and 24 h) to effectively sustain shoot structural expansion while maintaining a robust redox buffering capacity.

The upper-left quadrant functions as the thermally induced metabolic adjustment and phytate phosphorus retention domain, anchored by prominent vectors for S_Pphy and S_RSA. Immersion at elevated temperature (30 °C) systematically shifts genotypes into this space, ordered along the physiological response gradient by decreasing intensity: from the co-positioned apex activation exhibited by genotype L3 under prolonged exposures (co-clustering 3.5 and 3.6), through the configuration observable in genotype L1 at maximum immersion (1.6, exhibiting a lower PC1 coordinate combined with a higher PC2 elevation relative to 3.4), followed by intermediate exposures across genotype L3 and genotype L1 (1.5, 3.4, and 1.4), to moderate engagement shown by genotype L4 (4.5), and an initial defensive pivot manifested in genotype L2 under short exposure (2.4), extending to the quadrant boundary at 4.6. This spatial alignment indicates an active metabolic pivot away from primary biomass deposition, driven by a thermally induced shift towards the enhancement of radical scavenging capacity and a concurrent retention of phytate phosphorus reserves.

The lower-right quadrant represents the specialised photosynthetic pigment and secondary metabolite accumulation domain, defined by extended vectors for S_Chl *a*, S_Chl *b*, S_Chltot, S_tot phenol, and S_YP. Within this domain, genotype L2 demonstrates the highest secondary metabolite responsiveness, with its treatments distributed along the phenolic accumulation gradient from prolonged immersion at 20 °C (2.3) and the unprimed control (2.0), extending to the spatial displacement exhibited by genotype L4 under medium-term immersion (4.2). Positioned along these vectors, 2.3 and 2.0 represent the hallmark of phenolic and pigment accumulation, reflecting a metabolic priority towards secondary metabolite synthesis rather than active early shoot growth.

The lower-left quadrant marks the metabolic latency and suppressed vigour domain, situated opposite to the primary biomass expansion vectors. This domain defines a gradient of physiological unresponsiveness, sharply differentiating lower temperature-induced latency from elevated temperature-induced inhibition. Genotype 4 primarily reflects physiological inertia under lower temperature regimes (20 °C); its baseline control (4.0) alongside short and extended 20 °C immersion (4.1 and 4.3) remains at the lower boundary of this domain due to an absence of developmental triggering. Conversely, genotype L2 demonstrates a marked sensitivity to priming conditions, transitioning from lower-temperature latency at 20 °C (2.1 and 2.2) to pronounced thermal inhibition of shoot development under the elevated temperature regimes at 30 °C (2.5 and 2.6), which aligns with the constrained response observable in genotype L4 (4.4). Clustering within this domain highlights an inability of these specific genotype-treatment combinations to translate hydropriming stimuli into active shoot elongation and biomass deposition.

#### 2.2.3. Hierarchical Predictor Importance Governing Seedling Responses

**Figure 12:**
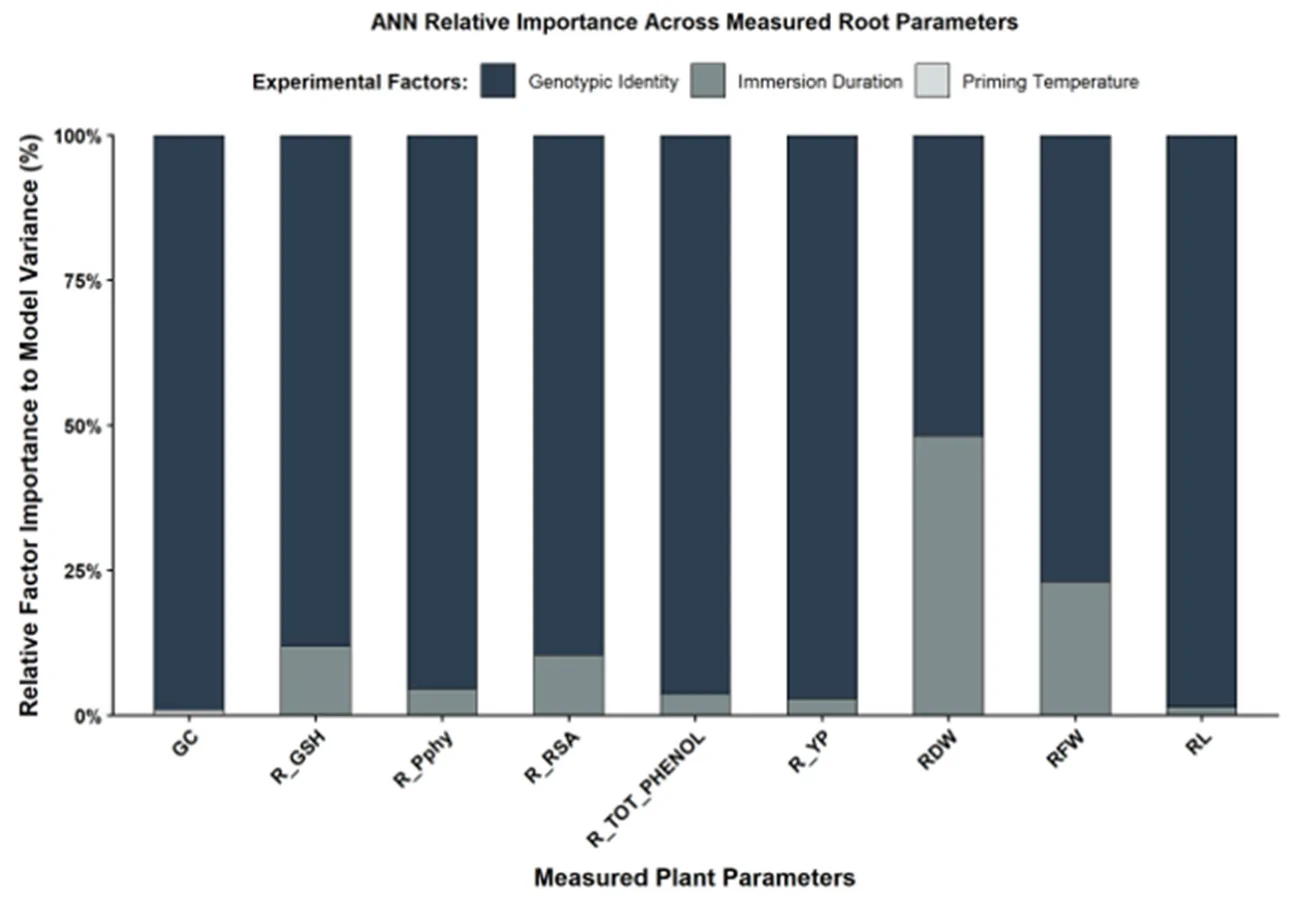
Relative factor importance across measured root parameters. Factor importance scores extracted from the artificial neural network model quantify the non-linear relative contribution of independent experimental factors to total model variance across individual root morpho-physiological parameters and metabolic markers. Parameter codes: GC—germination capacity; R_GSH—glutathione; R_Pphy—phytate phosphorus; R_RSA—radical scavenging activity; R_TOT_PHENOL—total water-soluble phenolics; R_YP—yellow pigment; RDW—root dry weight; RFW—root fresh weight; RL—root length. The stacked bar chart delineates the percentage contribution of three discrete experimental inputs—Genotypic Identity (dark blue-navy bars), Immersion Duration (mid-grey bars), and Priming Temperature (light grey bars)—to the total model variance explained via a multi-layer perceptron (MLP) model.

To evaluate the relative influence of individual experimental factors on root development and metabolism, an artificial neural network (ANN) analysis was conducted to partition model variance across each measured parameter (Figure 12, Tables S3 and S4). Genotypic identity emerges as the overwhelmingly predominant determinant of total variance across nearly all measured parameters, including germination capacity (GC), root length (RL), and the evaluated biochemical markers (R_GSH, R_Pphy, R_RSA, R_TOT_PHENOL, and R_YP), where its relative contribution continuously exceeds 75%. This overarching genetic dominance is selectively modulated by specific hydropriming parameters. Most notably, immersion duration operates as a highly critical secondary regulator for root biomass allocation, accounting for ~50% of the model variance for root dry weight (RDW) and ~25% for root fresh weight (RFW). Conversely, priming temperature exhibits a minimal direct contribution to variance partitioning across the measured root parameters, indicating that thermal adjustments within the evaluated windows trigger a highly uniform physiological plane that does not structurally reorganise the underlying factor-level hierarchy.

To further quantify these non-linear contributions of individual experimental factors and rank their systemic influence on root morpho-physiological parameters and metabolic markers, the artificial neural network (ANN) relative predictor importance analysis delineates a highly detailed, input-level importance hierarchy (Figure S1), closely echoing the physiological trajectory observed in the PCA biplot (Figure 11A). In full agreement with the factor-level variance partitioning, genotype identity emerges as the primary driver of overall variance in root parameters, predominantly outranking individual hydropriming regimes. Specifically, genotype L1 exhibits the highest relative importance score (~17.0), reflecting its continuous positioning across the high-vigour and redox-buffering domain. This is followed by treatment T0 (~11.3) and genotype L4 (~11.1), while genotype L3 (~9.7) and genotype L2 (~8.4) display intermediate-to-high scores.

Among the evaluated treatment factors, the unprimed baseline state (treatment T0) exerts the highest predictive weight (~11.3), underscoring a distinct baseline-dependent tissue sensitivity. This aligns with the spatial distribution in the PCA biplot, where the unprimed controls (2.0, 3.0, and 4.0) cluster at the bottom of the lower-right quadrant, whereas the control sample of genotype L1 (1.0) inherently resides within the upper-right high-vigour domain. Active hydropriming regimes, comprising treatments T5, T3, T2, and T1, demonstrate moderate predictive contributions (~8.1, ~7.0, ~6.0, and ~5.2, respectively), whereas treatments T6 and T4 register the lowest relative importance scores within the root system (~1.6 and ~1.1, respectively), indicating that elevated temperature conditions (30 °C) drive a uniform protective adjustment that reduces factor-level predictive resolution (Figure S1).

**Figure 13:**
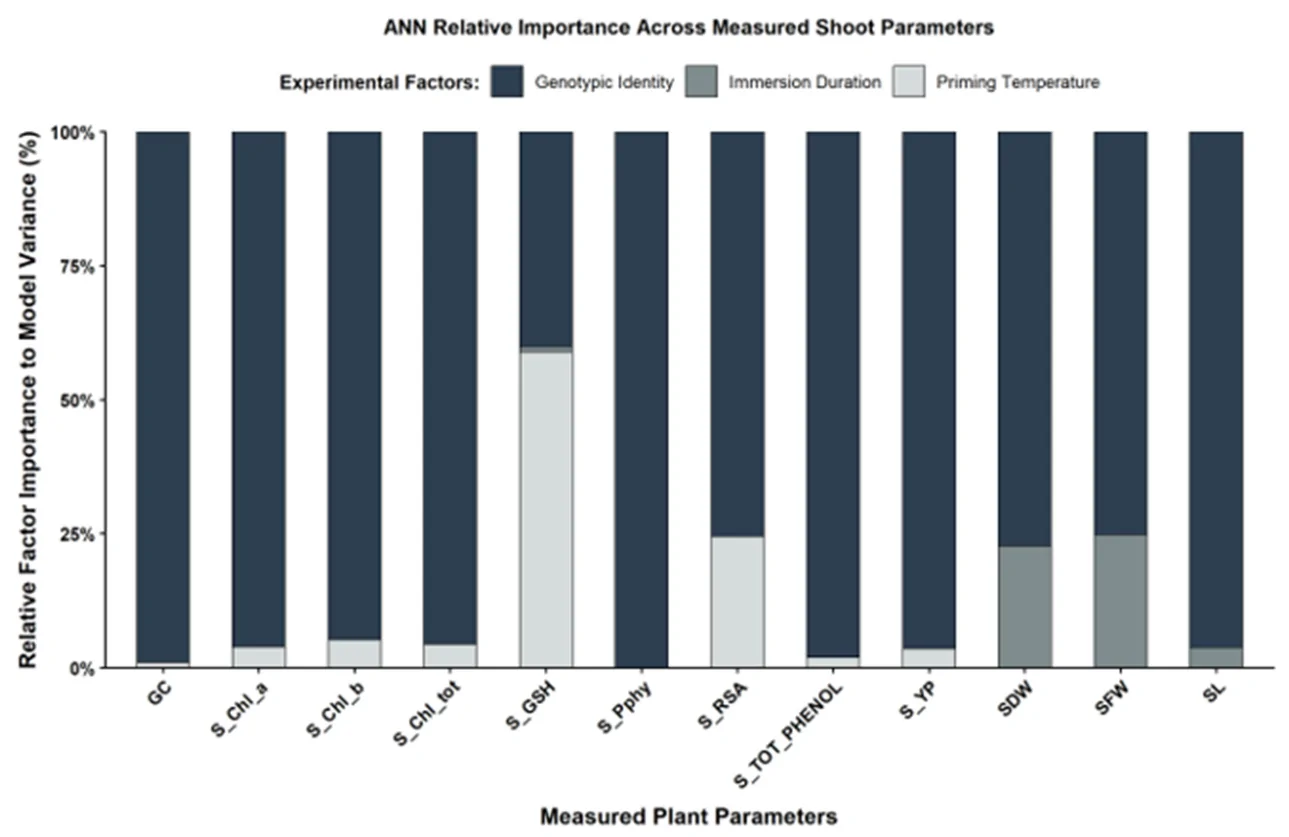
Relative factor importance across measured shoot parameters. Factor importance scores extracted from the artificial neural network model quantify the non-linear relative contribution of independent experimental factors to total model variance across individual shoot morpho-physiological parameters and metabolic markers. Parameter codes: GC (germination capacity); S_Chl_a (chlorophyll a); S_Chl_b (chlorophyll b); S_Chl_tot (total chlorophyll); S_GSH (glutathione); S_Pphy (phytate phosphorus); S_RSA (radical scavenging activity); S_TOT_PHENOL (total water-soluble phenolics); S_YP (yellow pigment); SDW (shoot dry weight); SFW (shoot fresh weight); SL (shoot length). The stacked bar chart delineates the percentage contribution of three discrete experimental inputs—Genotypic Identity (dark navy bars), Immersion Duration (mid-grey bars), and Priming Temperature (light grey bars)—to the total model variance explained via a multi-layer perceptron (MLP) model.

For shoot parameters, variance partitioning reveals highly distinct factor-level dynamics compared to the root system (Figure 13, Tables S3 and S5). Genotypic identity remains the primary determinant of model variance across the majority of measured traits, including germination capacity (GC), shoot length (SL), phytate phosphorus (S_Pphy), total water-soluble phenolics (S_TOT PHENOL), yellow pigment (S_YP), and the photosynthetic pigment pools (S_Chl_*a*, S_Chl_*b*, and S_Chl_tot). However, a profound metabolic divergence is observed for shoot glutathione content (S_GSH), where priming temperature operates as the dominant regulatory factor, accounting for ~59% of the total variance, whilst also contributing ~24% to the variance in shoot radical scavenging activity (S_RSA). Furthermore, immersion duration exhibits selective control over shoot biomass allocation, regulating ~25% of the variance in shoot fresh weight (SFW) and ~22% in shoot dry weight (SDW).

To further quantify these non-linear contributions at the input level, the relative predictor importance analysis ranks the specific influence of individual landraces and hydropriming regimes (Figure S2), mirroring the physiological clustering observed in the PCA biplot (Figure 11B). Genotype identity remains the predominant driver of overall variance, with genotype L3 demonstrating the highest relative importance score (~20.8), which aligns with its pronounced phenotypic plasticity across the optimal growth, vigour, and biomass accumulation domain. This is followed by treatment T3 (~10.0) and genotype L4 (~8.3). The elevated predictive weight of T3 confirms that extended immersion at lower temperatures (20 °C for 24 h) represents the critical physiological threshold for shoot metabolic differentiation. Genotypes L2 and L1 display intermediate scores (~7.2 and ~7.0, respectively).

Among the evaluated treatment factors, treatments T1 and T2 exhibit moderate predictive contributions (~4.3 and ~4.1, respectively), mirroring their role in maintaining moderate vigour accumulation along the positive PC1 axis under short- and medium-term immersion intervals at lower temperatures. Conversely, treatments T5 and T4, alongside the unprimed baseline state (T0), display lower predictive weights (~3.3, ~2.8, and ~2.4, respectively). Treatment T6 registers the lowest overall predictor importance score within shoot parameters (~0.3), indicating that exposure to the most intense regime (30 °C for 24 h) induces a generalised physiological suppression that diminishes its capacity to resolve genotype-specific performance differences (Figure S2).

## 3. Discussion

### 3.1. Hydration Dynamics and Physiological Thresholds for Seedling Homeostasis

The maintenance of a moderate baseline germination capacity across unprimed controls precludes structural-level tissue necrosis; instead, it indicates a finely tuned, physiological tolerance threshold regarding the conditions required for subsequent active seedling growth. Within a uniform dent morphotype shared by all four evaluated landraces, where the highly porous endosperm matrix characteristically accelerates hydraulic influx [43], the resulting germination capacity is determined by the individual cellular coordination and initial membrane integration status across the evaluated germplasm. As germination capacity reflects the embryo’s successful transition from metabolic quiescence to active autotrophic development, efficient hydration establishes intracellular water potential equilibrium, coupling moisture availability with repair pathways and macromolecular reserve mobilisation required to complete the germinative process [44]. Conversely, when seeds encounter prolonged immersion at lower temperature regimes, this environmental constraint triggers a highly distinct, homeostatic threshold response. According to Rolletschek et al. [45], such prolonged saturation shifts the cellular microenvironment toward restricted oxygen diffusion, establishing a state of transient intracellular hypoxia. Within the context of the evaluated germplasm, this mild, temporary hypoxia is proposed to operate as a protective, rate-limiting physiological mechanism that potentially precludes respiratory oxygen shock, thereby allowing sufficient time for orderly cellular membrane repair and subsequent downstream enzyme activation prior to radicle emergence [46]. Crucially, the efficiency of this homeostatic threshold is strictly genotype-dependent; for more responsive landraces, such as the genotype L3, this protective brake acts as an optimal eustress checkpoint—a trigger of adaptive, stimulatory defence and repair pathways—that maximises the final germination capacity, whereas for less responsive configurations with delayed signalling activation, like the genotype L2, the same interval marks the upper limit of metabolic tolerance prior to the onset of hypoxic stagnation, thereby limiting their germinative output. This species-specific dynamic window stands in stark contrast to other major cereals; for instance, wheat hydropriming typically peaks within a much narrower 8–12 h window at moderate temperatures (20–25 °C) before triggering cellular damage [16], whilst rice requires an extended 24–48 h saturation period due to a rigid hull morphology that restricts initial hydraulic influx [47]. For the evaluated maize landraces, the highly porous endosperm matrix of the dent morphotype creates a unique physiological plane, where the 24 h at 20 °C threshold successfully balances rapid initial hydration against the critical limits of hypoxic stagnation. Ultimately, this precise homeostatic alignment safely drives the germination capacity toward an optimised physiological plane, meaning that any variation in the final germination responses merely reflects a transient metabolic lag before achieving full metabolic alignment within the hydration-to-activation dynamics, thereby demonstrating a highly stimulatory hydropriming response.

During early seedling development, axial organ elongation drives the coordinated balance between meristematic cell division and cellular expansion—processes tightly modulated by internal resource partitioning and hormonal signalling. Specifically, short-term and medium-term immersion under elevated temperature regimes acts as a thermodynamic stimulus where the specific hydrothermal combination of hydration interval and thermal energy accelerates the rate of metabolic flux; this coordinated response potentially enhances membrane fluidity, thereby presumably boosting plasma membrane proton pump (H^+^-ATPase) activity to drive radicle cell elongation via acid-growth-induced cell wall extensibility [48]. In contrast, shoot elongation at these hydrothermal conditions is constrained by a physiological sink–source imbalance, wherein rapid radicle expansion establishes a dominant metabolic sink that prioritises anchorage and hydraulic acquisition while restricting reserve mobilisation to the embryonic shoot apices [49].

Beyond morphological dimensions, biomass accumulation provides an integrative measure of tissue hydration and structural reserve allocation, where fresh weight reflects cellular water retention and hydrostatic turgor pressure, whereas dry weight indexes net structural biomass derived from reserve mobilisation. The observed inhibitory effects on biomass stem directly from solute leaching and accelerated respiratory depletion during immersion, wherein rapid water entry across porous dent structures may force uncontrolled cellular rehydration prior to the complete restoration of membrane semi-permeability, resulting in the potential loss of low-molecular-weight soluble reserves via imbibitional damage [50]. Concurrently, intensified mitochondrial respiration potentially exhausts internal carbohydrate pools, leaving insufficient structural building blocks for anabolic cell wall deposition [41]. Nevertheless, the confinement of positive responsiveness to the specific medium-term immersion intervals indicates a distinct physiological threshold where optimal enzyme activation and membrane integrity restoration converge, facilitating efficient nutrient mobilisation and increased biomass accumulation.

### 3.2. Metabolic Adjustments and Antioxidant Defence

The metabolic configuration of young seedlings undergoes systemic biochemical shifts during the post-priming phase, transitioning from early reserve mobilisation through primary and secondary protective networks towards the functional development of the photosynthetic apparatus. Hydropriming regimes alter the temporal coordination of these sequential processes, imposing tissue-specific metabolic adjustments that dictate seedling establishment.

The enzymatic hydrolysis of phytic acid (PA) initiates the mobilisation of stored seed reserves during early germination [51,52]. As the primary intracellular reservoir of phosphorus and chelated mineral cations, PA operates as a critical metabolic regulator, with its phytate phosphorus (Pphy) fraction serving as a valuable indicator of reserve status; however, it reflects a steady-state pool size governed by the balance between synthesis, hydrolysis, and transport, rather than a direct measure of enzyme kinetics. Its hydrolysis by endogenous phytases is a fundamental prerequisite to liberate inorganic phosphate for ATP synthesis and plasma-membrane H^+^-ATPase activity [53,54]. The pronounced elevation in Pphy accumulation observed across tissues under hydropriming at the elevated temperature regimes indicates a severe kinetic uncoupling between accelerated initial imbibition and steady-state downstream phosphorylation. This thermal accumulation suggests that the elevated temperature regime severely hinders efficient reserve mobilisation, potentially creating an enzymatic bottleneck wherein accelerated water influx outpaces the rate of structural mineral utilisation, leaving essential phosphorus resources temporarily constrained within cellular compartments and presumably withholding energy pools from active cell wall deposition [55]. While this thermal accumulation may reflect impaired phytic acid degradation under heat stress, direct phytase activity measurements are ultimately required to substantiate this mechanism.

Concurrently, the rapid influx of oxygen upon rehydration activates mitochondrial respiration, generating an immediate ROS flux that threatens cellular integrity. The primary intracellular line of defence against this oxidative surge relies on the primary thiol redox buffering capacity of reduced glutathione (GSH)—a low-molecular-weight non-enzymatic antioxidant that preserves cellular redox homeostasis, maintains protein thiol groups in a functional state, and detoxifies lipid peroxides [56,57]. The substantially higher accumulation of GSH within the seedling root compared with the shoot underscores the heightened spatial sensitivity of root tissue to hydropriming-induced enhanced metabolic activity and thermal strain. This genotype-specific response—led by the consistent, high accumulation in the more responsive accessions, specifically the genotype L3, during short-term and medium-term immersion intervals under elevated temperature regimes—represents a robust, coordinated mode of cellular defence. Conversely, the continuous decline of GSH in genotypes exhibiting a more constrained capacity for thiol accumulation, such as genotype L1, which manifests early under lower-temperature treatments and persists across the remaining hydrothermal configurations, indicates a progressive exhaustion of the primary thiol pool, leaving cellular structures vulnerable to oxidative degradation [58,59].

When primary thiol lines are strained, seedlings deploy secondary protective systems consisting of water-soluble phenolics and a lipophilic yellow pigment pool. Phenolics act as potent secondary non-enzymatic antioxidants capable of chelating transition metals and reinforcing cell wall polymers [60], whereas the yellow pigment pool functions as a structural membrane stabiliser that scavenges singlet oxygen and shields lipid bilayers from photo-oxidation [61]. Hydropriming induces contrasting, tissue-specific reallocations of these compounds. The significant surge of total phenolics and yellow pigment in the root system under medium-term and prolonged immersion intervals at elevated temperatures represents a strategic metabolic prioritisation aimed at shielding radicle apical meristems from thermal and oxidative degradation, a phenomenon observed across all configurations but most prominently expressed in the highly responsive genotypes L3 and L4. Conversely, the sharp, severe decline of both parameters in the seedling shoot under these same intense hydrothermal regimes reflects a metabolic trade-off. This shoot-specific depletion demonstrates that elevated temperatures combined with extended immersion intervals selectively reorganise metabolic priorities, with priming temperature capturing exactly ~59% of the total model variance for shoot glutathione content and contributing ~24% to shoot radical scavenging activity. This regulatory shift presumably redirects isoprenoid and phenylpropanoid pathways preferentially toward the root axis, imposing a sink-driven depletion and a transient environmental vulnerability in the shoot tissue [62,63].

The collective efficiency of these individual hydrophilic and lipophilic antioxidant fractions culminates in total radical scavenging activity. Measured via the DPPH assay, RSA indexes the total tissue capacity to neutralise free radicals and prevent oxidative chain reactions. The uniform elevation of root RSA under all immersion intervals at elevated temperatures confirms that the coordinated accumulation of glutathione, phenolics, and yellow pigment successfully translates into an enhanced anti-oxidative shield within the root system [64]. However, the inconsistent response in the shoot—culminating in widespread reductions under a prolonged immersion interval—exposes a critical metabolic suppression. A prolonged immersion interval under lower-temperature regimes exhausts the total scavenging capacity of the shoot, indicating that extended hydration without thermal acceleration potentially fails to stimulate antioxidant enzymes adequately, leaving the shoot tissue in a state of compromised metabolic readiness [65].

The ultimate functional destination of this entire metabolic cascade is the synthesis of the photosynthetic pigment pool, which establishes the autotrophic potential and light-harvesting capacity of the seedling shoot. All immersion intervals at the elevated temperature regime cause a severe collapse across the entire photosynthetic pigment pool, demonstrating that thermal priming stress likely induces photo-oxidative degradation of plastids or severely disrupts chlorophyll biosynthetic pathways [66]. This physiological suppression confirms that rapid, thermally driven elongation of the root system occurs at the direct expense of shoot pigmentation, forcing the seedling to delay its autotrophic transition.

Conversely, the deployment of lower-temperature hydropriming regimes uncovers distinct, genotype-specific thresholds for photosynthetic development. The capacity of specific, more responsive genotypes, namely genotype L3, to achieve peak accumulation within the photosynthetic pigment pool under a prolonged immersion interval demonstrates precise metabolic alignment. At this optimal threshold, the steady, regulated mobilisation of reserves and the maintenance of a stable antioxidant status presumably allow the seedling to successfully bypass oxidative stress and channel available nutrients into the configuration of a robust light-harvesting apparatus [67]. This tissue-specific differentiation highlights that successful hydropriming relies on selecting protocols that stimulate post-storage metabolic reactivation and avoid the thermal downregulation of chlorophyll synthesis, thereby ensuring a swift and efficient transition to independent autotrophic growth.

### 3.3. Integrative Metabolic Plasticity and Genotypic Constraints of Seed Recovery and Subsequent Seedling Performance

The structural transition from seed quiescence to active seedling development relies on a strict, cell-level coordination that requires dynamic homeostatic plasticity and profound reconfiguration of embryonic metabolic pathways [68]. Under constitutive, unprimed conditions, this functional alignment operates under rigid, reserve-dependent control, where successful seed recovery and early seedling growth are strictly constrained by hydrolytic reserve breakdown and initial membrane stabilisation [52]. The strong linear alignment between unmobilised phytate reservoirs across root and shoot tissues confirms that nutrient retention in dent maize germplasm is governed by a tight, embryo-wide spatial integration [69]. Both phytate pools exert systemic control over the biosynthetic network—their intense negative correlation with the photosynthetic pigment complex provides definitive physiological proof that phytate consumption directly fuels *de novo* chlorophyll synthesis and plastid differentiation [70].

Within the radicle tissues, a strong positive co-dependency between GSH and total phenolics confirms a shared reliance of the nascent root on the pentose phosphate pathway for early metabolic reinforcement [71,72]. Crucially, this root-zone reinforcement operates via a strict cross-tissue metabolic trade-off: the intense inverse relationship between root GSH and shoot yellow pigment indicates that preferential allocation of redox resources to support radicle cell stabilisation temporarily suppresses pigment accumulation within the embryonic shoot [73]. This asymmetric biomass and resource allocation is quantitatively driven by specific priming parameters, with immersion duration accounting for ~50% of the total model variance for root dry weight and ~25% for root fresh weight, compared to ~22% and ~25% for shoot dry and fresh weights, respectively. Concurrently, within the shoot structure, a highly stringent inverse alignment between total phenolics and chlorophyll accumulation reveals a pronounced intra-tissue precursor competition, i.e., resource investment in cell wall phenolics restricts immediate assembly of the light-harvesting apparatus [63]. When this baseline state remains intact, radicle protrusion and initial biomass accumulation directly couple with constitutive radical scavenging activity and light-harvesting potential. Under conditions free of restrictive environmental pressures, this rigid coordination maximises structural anabolism, directly channelling reserve breakdown products into cell wall deposition and apical growth [74].

The deployment of hydropriming regimes at a lower temperature transforms this baseline dependency by transitioning metabolic control from raw reserve depletion to active primary thiol buffering and structural pigment organisation [24]. On a cellular level, a controlled imbibition rate relaxes the rigid spatial coordination of phytate pools, allowing the embryonic axis to execute a steady metabolic transition from strict reserve limitations. This shift reconfigures embryonic metabolic pathways, wherein primary thiol pools assume direct control over the cellular microenvironment. During the initial phase of hydration, the rapid activation of mitochondrial respiration and the upregulation of the phenylpropanoid pathway successfully couple early germination capacity and tissue elongation with enhanced shoot total phenolic accumulation, confirming the immediate induction of essential antioxidant protection while chloroplasts initiate chlorophyll biosynthesis [75]. As hydration progresses to an intermediate threshold, the physiological focus transitions towards radicle emergence; metabolic energy is preferentially directed into the incorporation of total phenolics within root cell walls to facilitate turgor-driven elongation and structural reinforcement [76], while the simultaneously observed retention of phytate phosphorus points toward a potential kinetic lag in *de novo* phytase synthesis during this short developmental phase [77].

The intense alignment between seedling biomass accumulation and the GSH pool under these optimal conditions reveals a crucial stabilisation phase. This metabolic reconfiguration reaches its physiological culmination under a prolonged immersion at the lower-temperature regime, which extends Phase II of germination. This extension provides embryonic cells with sufficient time for complete pre-germinative metabolic restoration and *de novo* synthesis of hydrolytic enzymes. Consequently, upon transitioning into Phase III, which is marked by radicle protrusion and subsequent coleoptile emergence, the accelerated activation of endogenous phytases facilitates efficient phytic acid cleavage and liberates inorganic phosphate to potentially support rapid ATP synthesis [77], establishing a conceptual energy surplus that is proposed to fuel the extensive expansion of the GSH pool across both vegetative structures [20]. Since this primary defence system effectively detoxifies ROS, the lipophilic shoot yellow pigment and newly synthesised chlorophyll components are completely preserved from photo-oxidative degradation and efficiently integrated into the thylakoid membranes [58,59]. Consequently, by successfully mitigating oxidative damage through this metabolically prioritised antioxidant shield, the embryonic cells conform to the expected model of metabolic alignment by channelling available resources into the configuration of a robust photosynthetic apparatus, effectively converting mobilised nutrients into functional light-harvesting pool synthesis and yellow pigment preservation [67].

Conversely, exposure to elevated temperature regimes prompts a progressive shift in metabolic control across immersion intervals, driving embryonic metabolic pathways from acute early strain and comprehensive exhaustion into an alternative survival strategy. Elevated temperature conditions severely disrupt the functional synergy between storage reserve mobilisation and structural early seedling growth. Thermally accelerated imbibition kinetics induce transient intracellular hypoxia as reported by Veselova et al. [78], potentially precipitating a profound metabolic disruption wherein downstream oxidative phosphorylation cannot sustain the energetic demands of rapid hydration. This putative impairment of cellular respiration is proposed to elicit severe homeostatic destabilisation, directly reflected in downstream alterations within subsequent biochemical cascades.

Short-term immersion imposes an immediate energy deficit that halts chlorophyll biosynthesis and arrests the transition of etioplasts to functional chloroplasts, driving a temporary inversion in photosynthetic co-linearity through antagonistic chlorophyll *a* and chlorophyll *b* positioning [66]. Concurrently, structural seedling elongation ceases, potentially reflecting a severe depletion within available ATP pools, which leads to a kinetic uncoupling between accelerated imbibition and downstream phosphorylation. To cope with this inferred metabolic uncoupling, the embryo manifests a stringent metabolic trade-off in precursor partitioning. This early stress response triggers an immediate accumulation of yellow pigment directly within the radicle apical meristems, forming a protective antioxidant shield against temperature-induced oxidative damage [79]. Furthermore, this metabolic shift strongly correlates with the retention of the unmobilised phytate phosphorus pool and its rigid alignment with root total phenolics.

Moreover, medium-term immersion-imposed intensified stress accelerates cellular exhaustion: the unmitigated retention of phytate phosphorus limits the availability of inorganic phosphate required for energetic processes, contributing to the decline in total chlorophyll development, while severe intracellular anoxia, as reported for maize under low-oxygen stress, is hypothesised to disrupt root-to-shoot nutrient translocation, thereby imposing a profound systemic constraint on the developing photosynthetic pigment complex [80]. Concurrently, this putative respiratory failure is proposed to drive root GSH into an intense inverse relationship with root total phenolics and shoot yellow pigment across both structures. This ultimate functional divergence confirms that severe hydrothermal stress forces the embryonic cells to permanently halt chloroplast development and divert all available intracellular resources into an immediate, compensatory survival response within the expanding tissue. In this context, our findings are in line with the classic concept of the plant dilemma, which dictates that growth and defense function as two strictly opposed, energy-consuming aspects of plant survival [81].

Crucially, this prolonged immersion bypasses the thermal inhibition and ensures cell viability under intense hydration limits by triggering an intricate secondary metabolic recovery. Within the root tissues, primary thiol pools re-establish dominant control, as the activated R_GSH pool drives a robust positive alignment with local radical scavenging activity (R_RSA) and chlorophyll *a* synthesis [32]. This coordination indicates that the embryonic cells successfully stabilise the baseline redox status within the elongation zone, effectively shielding radicle apical meristems from oxidative degradation. Simultaneously, within the shoot tissue, S_GSH and S_RSA form a coordinated, compensatory defence barrier that assumes direct control over biomass accumulation, indicating that autonomous activation of tissue-specific protective mechanisms successfully preserves newly synthesised chlorophyll *b* and total chlorophyll complexes from photo-oxidative breakdown [58]. This systemic redirection demonstrates that under extreme environmental pressure, the embryonic cells orchestrate precise, tissue-specific resource partitioning. Inductions of strong negative correlations with shoot total phenolics and yellow pigment pools across both vegetative fractions confirm that the embryo strategically suppresses secondary phenolic synthesis and unintegrated yellow pigment accumulation. This shift is highly indicative of a remarkably plastic survival strategy wherein cellular energy is strictly redirected to maintain the primary integrity of the cellular repair machinery and protect the vegetative meristems at the direct expense of developing light-harvesting systems. Such a strategic trade-off conceptually aligns with the mechanism of retrograde redox signalling from the plastids, which is proposed to actively suppress the synthesis and expansion of light-harvesting complexes to prevent photo-oxidative damage [82].

This functional seedling compartmentalisation expresses itself fully through the coordinated divergence of distinct tissue-specific developmental pathways. In the root tissue, the spatial organisation reveals a prominent axis of trade-off wherein structural biomass accumulation stands in direct physiological opposition to phytate phosphorus retention, indicating that biomass deposition remains strictly constrained by the primary energy requirements and structural building blocks necessary to sustain cellular expansion [83]. This root-specific dry matter partitioning is quantitatively validated by our predictive model, demonstrating that whilst immersion duration exercises a powerful control over radicle biomass determination—accounting for ~48% of the total model variance for root dry weight—its regulatory influence on root biochemical markers remains under 12%, confirming that dry matter deposition operates under separate physiological dynamics. Simultaneously, a separate metabolic cascade links active cell elongation directly to tissue-specific non-enzymatic antioxidant synthesis, implying that radicle expansion under shifting hydration dynamics is directly coupled with immediate localised scavenging capacity [84]. Conversely, the shoot tissue channels its primary developmental energy into a tightly linked coordination between photosynthetic pigment accumulation and cellular metabolic reconfiguration, separating these light-harvesting components from integrated seedling vigour and primary thiol homeostasis, which suggests a strict prioritisation mechanism governing precursor allocation between structural chloroplast differentiation and defensive redox shielding [56]. This shoot-specific prioritisation is mathematically substantiated by the fact that priming temperature dictates up to ~59% of the total variance for shoot glutathione allocation, while exerting a negligible influence on vegetative shoot elongation. The integration of such predictive neural network frameworks to model non-linear physiological pathways aligns with the expanding application of digital technologies and intelligent algorithms in modern precision agriculture to decode multifaceted plant stress responses [85].

When mapped across these tissue-specific developmental trajectories, the performance profiles partition into distinct functional domains that delineate the homeostatic strategy of each genotype. The domain of optimal vigour and biomass accumulation represents the functional anabolic trajectory, wherein a controlled imbibition rate successfully pairs substantial cell wall deposition and rapid structural tissue expansion with a robust primary thiol and redox buffering capacity, a physiological pathway fully exemplified by genotypes L1 and L3. More responsive genotypes sustain this high-vigour pathway natively, demonstrating an exceptional physiological plasticity that maintains an active metabolism and strong cell turgor pressure across diverse shifting hydration dynamics [86]. Less responsive genotypes require prolonged immersion intervals under the lower-temperature regime to overcome intrinsic metabolic latency and enter this high-vigour plane, indicating that less responsive germplasm faces higher intracellular constraints related to metabolic repair windows and delayed *de novo* enzyme activation during the early phases of seed hydration [87].

When environmental parameters shift towards elevated-temperature regimes, the metabolic pathways transition into the domain of enhanced cell elongation and secondary metabolic defence. Within the root, this reflects a highly coordinated metabolic redirection wherein resources divert away from net weight accumulation and channel into rapid axial elongation, coupled with a massive upregulation of total water-soluble phenolics, yellow pigment, and radical scavenging mechanisms, confirming the preferential activation of phenylpropanoid and isoprenoid pathways locally within the radicle [63,88,89]. This localised response shields the radicle apical meristems from thermal and oxidative degradation, demonstrating that lipophilic yellow pigment functions to stabilise membrane bilayers against temperature-induced fluidity while soluble phenolics reinforce the extending cell wall matrix, a protective phenomenon observable in genotypes L3 and L4 [90]. Within the shoot tissue, this same thermal stimulus forces a complete metabolic pivot away from primary vegetative growth and chlorophyll synthesis, systematically channelling metabolic energy into defensive antioxidant mobilisation and an intense unmitigated retention of reserves, validating a profound tissue-specific energetic uncoupling that is prominent in genotypes L1 and L3. This systemic arrest is strongly consistent with the thermal downregulation of endogenous phytase transcription under hydrothermal strain [91], while simultaneously conforming to the expected model of transient tissue hypoxia that triggers the induction of defensive antioxidant mechanisms [92] at the absolute expense of light-harvesting biosynthetic pathways. This tissue-specific partitioning implies that under thermal strain, the seedling prioritises immediate localised survival and accepts a transient downregulation of its light-harvesting potential.

In sharp contrast, the limits of homeostatic plasticity demonstrate the consequences of suboptimal activation and pronounced thermal strain. The quiescent baseline and metabolic decoupling domain define a state of physiological inertia typically observed under lower-temperature regimes without thermal acceleration, as observable in genotypes L3 and L4. Under these conditions, the seedling preserves basic structural attributes but fails to receive a sufficient tissue-specific metabolic trigger to initiate rapid tissue expansion, leading to a distinct physiological uncoupling wherein early germination events remain completely disconnected from active structural root development, demonstrating that lower-temperature profiles fail to provide the thermodynamic stimulus required to activate critical enzymatic activation thresholds, which subsequently implies that the overall metabolic dynamics remain below the critical threshold necessary to trigger the transition into Phase III developmental expansion [93,94].

This state of developmental stagnation deepens within the domain of reserve retention and metabolic latency, which stands in direct physiological opposition to the pathways of primary biomass expansion. Here, the metabolic pathways enter a state of severe latency characterised by a total failure of phytate cleavage and a complete loss of metabolic responsiveness across all evaluated parameters, confirming that essential mineral and organic resources remain structurally constrained within the cellular matrix. Under lower-temperature regimes, this domain captures developmental dormancy due to an absence of an exogenous triggering stimulus, as exhibited by genotype L4 [95]. However, under extreme conditions, this domain transitions into a zone of pronounced thermal inhibition, wherein less responsive genotypes experience a severe functional impairment of cellular respiration and a total collapse of shoot development, as shown in genotype L2. The distinct separation of these trajectories provides mechanistic proof that when the shifting hydration dynamics are misaligned with the internal rate of enzymatic activation, the seedling enters a state of structural and tissue-specific metabolic suppression that permanently blocks its autotrophic transition, revealing that accelerated water influx combined with persistent thermal strain induces an irreversible ROS accumulation that exhausts the primary glutathione pool, thereby potentially initiating lipid peroxidation and arresting mitogenesis within the embryonic shoot axis [96].

To understand the underlying regulatory mechanisms wherein successful pre-germinative seed recovery induces enhanced seedling performance, evaluating the entire developmental network is essential to establish whether the final phenotypic outcome is governed by intrinsic genetic programming or driven by external hydrothermal stimuli. In this regard, a systemic evaluation establishes that intrinsic genotypic architecture operates as the primary determinant of total developmental plasticity across both vegetative structures [97]. The genetic predominance of particular accessions—namely genotype L1 within the root tissue and genotype L3 within the shoot tissue—demonstrates that the underlying genetic identity defines the strict operational boundaries of structural reconfiguration and homeostatic buffering. Physiologically, this genotypic dominance is hypothesized to reflect an inherent capacity to maintain mitochondrial membrane integrity under hydrothermal pressure, which is proposed to prevent electron leakage and support a stable ATP supply required for active cellular division, radicle elongation, and subsequent biomass accumulation [75].

Furthermore, the dominant regulatory influence of the unprimed baseline state in roots confirms a distinct baseline-dependent tissue sensitivity, implying that the initial metabolic condition and embryonic membrane integrity preserved during ex situ storage dictate the substrate thresholds for subsequent reactivation [98]. Crucially, when hydration is properly managed through lower-temperature hydropriming, the radicle tissues successfully escape the physical disruptions, accelerated imbibition damage, and the respiratory restriction and oxygen limitation typically associated with elevated temperatures [99]. This metabolic preservation presumably allows for unhindered oxidative phosphorylation, which appears to support the timely mobilisation of phytate phosphorus reserves [100]. Consequently, this energetic efficiency fuels the immediate biosynthesis and accumulation of GSH and total phenolics, effectively establishing a robust non-enzymatic antioxidant shield that mitigates early oxidative stress during radicle elongation [24].

In the shoot, the critical role played by prolonged immersion at lower-temperature regimes (24 h at 20 °C) confirms that this precise threshold appears to operate as a critical physiological turning point for embryonic shoot expansion and chloroplast differentiation, potentially accelerating the metabolic activation of hydrolytic enzymes to drive pigment retention and biomass deposition [101,102]. By maintaining imbibition at this optimal 20 °C threshold, the embryonic tissues successfully maintain membrane integrity and presumably limit excessive ROS accumulation. This metabolic stability conceptually aligns with the protective role of heat shock proteins (HSPs) and late embryogenesis abundant (LEA) proteins, which are proposed to systematically stabilise the emerging photosynthetic apparatus [103]. This protective mechanism potentially prevents the degradation of the yellow pigment and chlorophyll pools, while concurrently maintaining cellular redox homeostasis through the well-regulated coordination of GSH pools and phenolic compounds within the developing shoot structures [104].

### 3.4. Relevance to Ex Situ Conservation: Methodological Boundaries and Limitations

According to the framework established by Fabrissin et al. [105], integrating pre-sowing hydration protocols into ex situ conservation involves a critical physiological trade-off between accelerated germination vigour and long-term seed longevity. In routine gene bank operations, managing the risk of accelerated decline during the back-drying phase represents a critical physiological bottleneck. Consequently, the hydrothermal priming protocol presented herein is strictly recommended as a targeted, pre-sowing intervention for specific regeneration plots, rather than a method for long-term re-storage. Because the desiccation stress associated with post-priming back-drying can compromise cellular integrity, restoring these primed accessions under standard medium-term gene bank conditions (at +4 °C) is highly discouraged until long-term viability post-back-drying is systematically quantified.

The application of this protocol must be evaluated based on the specific requirements of individual seed lots. While hydrothermal priming provides exceptional cost-efficiency by relying solely on water and precise temperature control, its routine application is bounded by the high inter- and intra-specific variability of seed germplasm. Our experimental matrix was restricted to four distinct accessions belonging to a single morphotype. Given that pericarp permeability, imbibition kinetics, and hydrothermal sensitivity vary significantly across disparate maize groups, implementing this protocol requires prior, morphotype-specific optimisation. Furthermore, whilst our investigation was specifically designed to capture the cumulative physiological and metabolic profiles of fully established, 7-day-old seedlings, a comprehensive operational optimisation of this priming protocol must also integrate early-stage germination kinetics—specifically the germination rate, synchrony, and mean germination time—which remain highly contingent upon genotypic variations. Therefore, this protocol constitutes a conceptual baseline for targeted interventions rather than a universal standard. To transition this experimental optimisation into actionable conservation strategies, future investigations must delineate the exact operational boundaries between metabolic rejuvenation and physiological decline during the regeneration of low-viability germplasm.

## 4. Materials and Methods

### 4.1. Plant Materials

The Maize Research Institute Zemun Polje (MRIZP) Gene Bank active maize collection is maintained under medium-term storage conditions at +4 °C, an approximate relative humidity of 40%, and a seed moisture content of 11%, in strict compliance with international guidelines for the effective management of germplasm collections [106]. This extensive repository comprises 5806 accessions, out of which 2217 are traditional local landraces, thereby representing a highly valuable and diverse reservoir of genetic resources.

Routine viability monitoring of the stored accessions revealed that germination in certain accessions had declined below the prescribed gene bank standards, falling below the critical threshold of 85%. Consequently, for the present study, four traditional landraces originating from diverse agro-ecological groups were selected to evaluate the efficacy of pre-sowing hydropriming in stimulating post-storage germinative reactivation and subsequent seedling development. Information regarding the evaluated germplasm is summarised in Table 2. Prior to the experiment, seeds were manually screened to ensure physical uniformity, and any broken, shrivelled, or mechanically damaged kernels were eliminated.

### 4.2. Experimental Design and Hydropriming Protocols

To evaluate the interactive effects of genotype, priming temperature, and immersion intervals, the hydropriming treatments were structurally designated based on their respective parameters as T1 (20 °C, 8 h), T2 (20 °C, 16 h), T3 (20 °C, 24 h), T4 (30 °C, 8 h), T5 (30 °C, 16 h), and T6 (30 °C, 24 h). For each accession, an unprimed, dry seed lot directly retrieved from storage was maintained without water immersion to serve as the baseline control (T0). For every individual treatment regime and accession, assays were performed utilising three independent biological replicates consisting of 50 physically uniform seeds each (*n* = 3).

Prior to treatment, seeds were surface-sterilised with a 1% (*v*/*v*) sodium hypochlorite solution for 10 min, followed by thorough rinsing with distilled water. Seeds were subsequently submerged in individual glass vessels containing exactly 200 mL of sterile distilled water, ensuring a constant water-to-seed ratio and complete immersion under dark conditions in thermostatic chambers configured to either 20 ± 0.5 °C or 30 ± 0.5 °C according to the designated immersion intervals (8 h, 16 h, or 24 h).

Upon completion of each specified time interval, primed seeds were retrieved, gently blotted with lint-free filter paper to eliminate surface-adhered moisture, and immediately subjected to a precise, controlled dehydration (post-priming back-drying) process. Seeds were spread in a single layer on absorbent analytical paper sheets and maintained under controlled ambient laboratory conditions (20–22 °C, ~50% RH). Seed lots were periodically monitored and weighed until reverting precisely to their initial baseline weight, corresponding to a moisture content equilibrium state of ~11%. This dehydration phase effectively arrested active metabolic progression and prevented premature radicle protrusion, while successfully preserving the metabolic preconditioning state and establishing a stable priming memory prior to subsequent germination testing and biochemical analyses.

### 4.3. Germination Testing and Seedling Growth Metrics

Following the completion of the hydropriming regimes and post-priming back-drying procedures, standard germination tests were conducted on all experimental seed lots in strict accordance with International Seed Testing Association (ISTA) Rules [107] using the between-paper method to evaluate germination capacity (GC%). The primed and unprimed control seeds were arranged uniformly on pre-moistened germination papers, rolled, and placed into sealed polyethylene bags to maintain constant relative humidity. Incubation was conducted in a precision climate chamber (MLR-352H, Panasonic, Osaka, Japan) configured to an alternating temperature regime of 20/30 °C under a strict 8/16 h light/dark photoperiod (with a light intensity of 1600 lx) for 7 days.

Germination evaluation followed strict ISTA morphological criteria based on the synchronised growth of essential embryonic structures. Seedlings exhibiting structural abnormalities (such as deformed coleoptiles, spiral mesocotyls, or stunted primary roots) were classified as abnormal and excluded from the total counts. Normal seedlings were identified by the emergence of the first foliage leaf from the coleoptile and a well-developed root system comprising the primary root and lateral secondary roots. Following ISTA protocols, GC (representing the potential of seeds to develop into normal seedlings) was evaluated on the 7th day (final count). The percentage of GC was calculated according to Zhang et al. [108]:

(1) $$\mathrm{GC}\;(\%)\;=\;(\frac{N_{\mathrm{norm}}}{N_{\mathrm{total}}})\;\times \;100$$

where *N_norm_* represented the number of normally germinated seedlings evaluated on Day 7, and *N_total_* was the total number of tested seeds per replicate (*N_total_* = 50).

Immediately following the final germination count on the 7th day, ten representative normal seedlings per replicate were randomly selected for morphometric and biomass evaluations. Root length (RL) was measured from the scutellar node to the tip of the primary root, while shoot length (SL) was recorded from the scutellar node to the apex of the longest foliage leaf. Seedlings were subsequently dissected into root and shoot fractions to evaluate biomass allocation. Root fresh weight (RFW) and shoot fresh weight (SFW) were immediately determined on a digital analytical balance. Subsequently, the separated root and shoot tissues were oven-dried at 40 °C for 12 h until reaching a constant weight before being re-weighed to record root dry weight (RDW) and shoot dry weight (SDW).

### 4.4. Biochemical Assays

Biochemical evaluations were conducted on fresh tissue samples pooled from the ten randomly selected normal seedlings per replicate (previously subjected to morphometric and fresh biomass measurements), which were harvested from the separated roots and shoots of 7-day-old maize seedlings across all treatment regimes and controls. For each biological replicate, the harvested fresh tissues were thoroughly homogenised into a uniform matrix, and separate aliquots of this fresh homogeneous tissue sample were immediately weighed for each specific assay described below to ensure biological consistency across all measurements. To eliminate confounding effects resulting from variable tissue hydration following hydropriming, the content of biochemical markers was calculated and expressed on a dry weight (DW) basis using the tissue moisture content determined from parallel oven-dried representative seedling samples comprising the remaining normal seedlings from the same biological replicate. All assays were performed in three independent biological replicates (*n* = 3), each conducted in three technical replicates. Centrifugation steps across all procedures were executed at 14,000× *g* for 15 min at 4 °C using a Model Velocity 18R Versatile Centrifuge equipped with a TA15-24-2 rotor (Dynamica Scientific, Livingston, UK), and spectroscopic measurements were performed using a Biochrom Libra S22 UV/Vis spectrophotometer (Biochrom, Cambridge, UK).

The contents of phytate phosphorus (Pphy) and total water-soluble phenolics were quantified from identical aliquots of fresh tissue (200 mg) homogenised and extracted with 1.5 mL of double-distilled water, which served as the common aqueous extraction matrix. Following centrifugation to secure clear supernatants, the Pphy content was quantified directly from the aqueous extract in accordance with the colorimetric method described by Dragičević et al. [109] utilising the Wade reagent. Aliquots of the water extract were reacted with the Wade reagent, which comprised 0.03% FeCl_3_**·**6H_2_O and 0.3% sulfosalicylic acid, and the reduction in the intensity of the pink colour, generated by the reaction between ferric ions and sulfosalicylic acid, was recorded at λ = 500 nm. Total glutathione (GSH) content (representing the total acid-soluble sulfhydryl pool) was estimated spectrophotometrically according to the protocol proposed by Sari-Gorla et al. [110], utilising fresh tissue aliquots (200 mg) extracted from the same homogenised sample pool with 1.5 mL of 5% (*w*/*v*) trichloroacetic acid (TCA) to ensure protein precipitation. For this assay, aliquots of the clear TCA supernatant were treated with 0.2 M potassium phosphate buffer (pH = 8.0) and 10 mM 5,5′-dithio(2-nitrobenzoic acid) (DTNB) dissolved in the buffer matrix. The reaction between DTNB and the thiol groups quickly yields the yellow-coloured 5-thio-2-nitrobenzoic acid (TNB) product, after which the absorbance was recorded at λ = 415 nm. Quantitative data for both biochemical markers were calculated using their respective standard calibration curves constructed with sodium phytate and reduced glutathione, respectively, and the final results were expressed on a dry weight basis as mg of Pphy and μg of GSH per g of dry matter (mg g^−1^ d.m. and μg g^−1^ d.m., respectively).

The quantification of total water-soluble phenolics was conducted using the modified Prussian blue method described by Simić et al. [111] from the same homogenised fresh tissue matrix. In brief, identical aliquots of fresh tissue (200 mg) were homogenised with 1.5 mL of double-distilled water. Following centrifugation to yield a clear supernatant, aliquots of this extract were reacted with 0.05 M FeCl_3_**·**6H_2_O dissolved in a 0.1 M HCl matrix, followed by the immediate addition of 0.008 M K_3_Fe(CN)_6_ to initiate the chemical formation of the stable Prussian blue complex. Following a 25-min incubation period at room temperature in the dark, the absorbance was recorded at λ = 722 nm. Quantitative data were extrapolated from a ferulic acid standard calibration curve, and the results were expressed on a dry weight basis as μg of ferulic acid equivalent per g of dry matter (μg FAE g^−1^ d.m.).

The contents of yellow pigment (YP, representing the total lipophilic carotenoid pool) and photosynthetic pigments, including chlorophyll *a* (Chl *a*), chlorophyll *b* (Chl *b*), and total chlorophyll (Chl_tot_), were determined spectrophotometrically from the fresh tissues in accordance with the specific absorption coefficient equations proposed by Lichtenthaler and Buschmann [112] without external standard curves. In brief, identical aliquots of fresh tissue (200 mg) were further homogenised and extracted with 80% (*v*/*v*) aqueous acetone, which served as the extraction solvent matrix. To facilitate cellular disruption and prevent enzymatic pigment degradation, a small quantity of quartz sand and calcium carbonate (CaCO_3_) was introduced during the homogenisation step. Following centrifugation at 14,000× *g* for 15 min at 4 °C to secure a completely clear supernatant, absorbance values were recorded at λ = 440 nm for the YP estimation, in conjunction with the chlorophyll absorption maxima at λ = 662 nm for Chl *a* and λ = 644 nm for Chl *b*, utilising 80% (*v*/*v*) acetone as the blank matrix. Quantitative data were calculated using the corresponding extinction coefficients, and the results were expressed on a dry weight basis as mg of the respective pigment per g of dry matter (mg g^−1^ d.m.).

The total radical scavenging activity (RSA) of the root and shoot tissues was determined using the 2,2-diphenyl-1-picrylhydrazyl (DPPH) free radical assay in accordance with the method proposed by Brand-Williams et al. [113]. In brief, aliquots of the fresh tissue extracts, obtained via extraction with 70% (*v*/*v*) aqueous acetone, were mixed with a 0.1 mM DPPH radical solution prepared in methanol. The reaction mixture was vortexed and subsequently incubated in the dark at room temperature for 30 min to facilitate the reduction of the free radicals. Following the incubation period, the decrease in absorbance was recorded spectrophotometrically at λ = 517 nm against a pure solvent blank to zero the instrument, where an untreated control mixture containing DPPH and the extraction solvent served as the reference point for estimating total radical scavenging performance. The radical scavenging activity was calculated and expressed as the percentage of DPPH radical inhibition based on the absorbance difference between the blank control and the sample containing the plant extract.

### 4.5. Statistical Analysis

The consolidated dataset was statistically analysed using a two-way factorial analysis of variance (ANOVA) within a randomised complete block design (RCBD) to evaluate the main effects of genotype (G), hydropriming treatment (T), and their interaction (G × T). The *F*-statistics, associated significance levels, and experimental precision expressed as the coefficient of variation (CV, %) for all evaluated physiological and biochemical traits are presented in Table S1. Significant differences between treatment and genotype means were determined by Tukey’s honestly significant difference (HSD) test, with differences at the *p* ≤ 0.05 probability level considered significant.

To avoid pseudoreplication, correlation matrices and multivariate models were executed on a dataset comprising *n* = 12 independent biological observations (4 landraces × 3 biological replicates) per hydropriming treatment. Linear relationships among seedling growth parameters and biochemical markers were evaluated via Pearson’s correlation matrices, with the statistical significance of Pearson’s r determined at *df* = *n* − 2 = 10 degrees of freedom. Furthermore, a Principal Component Analysis (PCA) was performed on the matrix of mean values for the *N* = 28 Genotype × Treatment (G × T) combinations to assess structural patterns and identify primary sources of variation utilising the R software environment. Prior to conducting the PCA, all variables were standardised using z-score transformation to eliminate the effects of different measurement units among the morpho-physiological and biochemical parameters. Additionally, 95% confidence ellipses were constructed based on the bivariate normal distribution of the factor scores corresponding to these *N* = 28 G × T interaction means. The contributions of variables (%) to the principal components (PCs) within the integrated PCA biplots are provided in Supplementary Table S2.

To capture complex non-linear interactions and assess the relative contribution of experimental factors, an artificial neural network model was constructed separately for root and shoot datasets. The experimental factors—comprising genotypic identity (the four evaluated landraces) and experimental treatments (the unprimed control T0 and hydropriming regimes T1–T6)—served as input categorical predictors, whilst the evaluated morpho-physiological and biochemical parameters constituted the model outputs, formally designated as target outputs. Architecturally, the neural estimation was deployed utilizing a single-hidden-layer Multilayer Perceptron (MLP) framework structurally constrained to exactly three hidden neurons (size = 3) to prevent over-parameterisation and ensure optimal generalisation given the compact nature of the dataset (*N* = 28 treatment-genotype means), as formally detailed in Supplementary Table S3. Prior to network execution, all continuous variables underwent automated Z-score standardisation mapping to achieve variance equilibrium among parameters.

Relative predictor importance scores were extracted from the trained root and shoot models to rank the systemic influence of landrace divergence versus treatment effects across both tissue fractions via non-linear relative importance partitioning (varImp). To guarantee complete mathematical reproducibility, a fixed random initialization seed (set.seed = 42) was enforced. The prediction accuracy and internal stability of the networks were rigorously evaluated through a 4-fold cross-validation scheme (k = 4), which iteratively partitioned the dataset into a training subset of exactly 21 observations (75%) and an independent testing subset of 7 observations (25%) per fold. Synaptic weights were tightly regulated via an L2 regularisation penalty (weight decay = 0.05) to suppress stochastic noise, and final model validation was quantified using the Coefficient of Determination (R^2^), Root Mean Square Error (RMSE), and Mean Absolute Error (MAE). The resulting performance metrics and relative predictor importance percentages are fully reported in Supplementary Table S4 for the root system and Supplementary Table S5 for the shoot system, with their corresponding factor variance partitions visually illustrated in Supplementary Figure S1 and Supplementary Figure S2, respectively.

All statistical computations, model fittings, multivariate modelling, and data visualisations were executed within the R software environment (R version 4.5.3) [114].

## 5. Conclusions

Given that large-scale germplasm collections manage thousands of maize accessions that lack comprehensive biochemical characterisation regarding pericarp structure and endosperm texture, and inherently exhibit variable water imbibition dynamics, the deployment of highly individualised protocols is practically unfeasible. In response to this challenge, our findings delineate that exposure to elevated temperatures of 30 °C should be stringently avoided, as this treatment severely constrains early vegetative development. Conversely, short-term hydration windows at lower temperatures remain insufficient to fully overcome structural latency. The evaluation of accessions from diverse agro-ecological origins effectively demonstrates that hydropriming, set at a prolonged immersion period of 24 h strictly maintained at 20 °C, provides a highly effective and operationally viable stimulatory framework. By safely stimulating post-storage germinative reactivation and seedling development in the investigated germplasm under controlled conditions, this specific protocol increases early seedling growth and protects the developing photosynthetic apparatus during the critical heterotrophic-to-autotrophic transition. However, given the limited number of genotypes evaluated in this study, the observed response patterns must be interpreted as trait- and genotype-specific. Further studies under dynamic field conditions across wider genetic diversity panels covering different endosperm textures and ecotypes are required to monitor subsequent plant growth until maturity and to fully evaluate the efficiency of this pre-sowing treatment during routine germplasm regeneration.

## Figures and Tables

**Table 1:** Principal component analysis (PCA) factor loadings of integrated morpho-physiological and biochemical profiles in seedling (**A**) root and (**B**) shoot tissues across unprimed and hydroprimed maize landraces.

**(A)**		
**Seedling Root**		
**Parameters**	**PCA1**	**PCA2**
Eigenvalues	3.15 ^1^	2.76
Variance (%)	34.98	30.94
Cumulative variance (%)	34.98	65.92
Traits (Factor Loadings)		
Germination capacity (GC)	0.634	0.539
Root length (RL)	−0.289	**0.732**
Root fresh weight (RFW)	**0.849**	0.402
Root dry weight (RDW)	**0.822**	0.404
Root phytate phosphorus (R_Pphy)	**−0.700**	−0.029
Root total glutathione (R_GSH)	0.172	0.664
Root total phenolics (R_tot phenol)	−0.582	**0.710**
Root yellow pigment (R_YP)	−0.599	**0.708**
Root radical scavenging activity (R_RSA)	−0.233	0.431
**(B)**		
**Seedling Shoot**		
**Parameters**	**PCA1**	**PCA2**
Eigenvalues	6.38	3.58
Variance (%)	53.18	29.82
Cumulative variance (%)	53.18	83.00
Traits (Factor Loadings)		
Germination capacity (GC)	0.503	**0.700**
Shoot length (SL)	0.624	0.660
Shoot fresh weight (SFW)	0.603	**0.722**
Shoot dry weight (SDW)	0.572	**0.728**
Shoot phytate phosphorus (S_Pphy)	**−0.791**	0.369
Shoot total glutathione (S_GSH)	0.495	**0.725**
Shoot total phenolics (S_tot phenol)	0.686	−0.573
Shoot yellow pigment (S_YP)	**0.750**	−0.614
Shoot radical scavenging activity (S_RSA)	**−0.794**	0.304
Shoot chlorophyll *a* (S_Chl *a*)	**0.937**	−0.184
Shoot chlorophyll *b* (S_Chl *b*)	**0.881**	−0.257
Shoot total chlorophyll (S_Chl_tot_)	**0.927**	−0.203

Factor loadings > |0.700| are shown in bold. ^1^ Principal components retained according to Kaiser’s criterion (eigenvalue > 1); minus signs (−) denote negative correlations.

**Table 2:** Passport data, historical storage viability profile, and kernel morphotypes of the evaluated traditional maize landraces. Legend: GO (CS)—Geographic Origin (Collection Site); AG—Agro-ecological Group; YLR—Year of Last Regeneration; SD—Storage Duration (years); IGC—Initial Germination Capacity (%); CGC—Control Germination Capacity (%); ET—Endosperm Texture; BIH—Bosnia and Herzegovina; HR—Croatia; SRB—Serbia; SLO—Slovenia.

Sample	Accession ID	Vernacular Name	GO (CS)	AG	YLR	SD	IGC (%)	CGC (%)	ET
L1	MRIZP01877	Cadjo	BIH	Bosnian early dents	2002	25	100	81	dent
L2	MRIZP01917	Osmak	HR	Eight-rowed soft dents	2002	25	97	68	dent
L3	MRIZP01766	Zlatni zuban	SRB	Dents of USA Corn Belt	2002	25	99	77	dent
L4	MRIZP01986	Avgustnik zuti	SLO	Dents of southern USA	2002	25	98	64	dent

## Data Availability

The data presented in this study are available in the article.

## Supplementary Materials

The following supporting information can be downloaded at https://www.mdpi.com/article/10.3390/plants15213479/s1, Table S1: Two-way analysis of variance (ANOVA) *F*-statistics, significance levels, and coefficients of variation (CV) for the evaluated morpho-physiological and biochemical traits in local maize landraces under hydropriming treatments; Table S2: Contribution of variables (%) to the first two principal components (PC1 and PC2) for: (a) seedling root and (b) seedling shoot; Table S3: Artificial Neural Network architecture and data partitioning; Table S4: Performance metrics and relative predictor importance (%) of the Multi-Layer Perceptron (MLP) neural network models governing maize root responses under hydropriming regimes; Table S5: Performance metrics and relative predictor importance (%) of the Multi-Layer Perceptron (MLP) neural network models governing maize shoot responses under hydropriming regimes; Figure S1: Relative predictor importance for root parameters; Figure S2: Relative predictor importance for shoot parameters.

## Abbreviations

The following abbreviations are used in this manuscript:

GC	germination capacity
RFW	root fresh weight
RDW	root dry weight
RL	root length
SFW	shoot fresh weight
SDW	shoot dry weight
SL	shoot length
Pphy	phytate phosphorus
GSH	reduced glutathione
YP	yellow pigment
DPPH	2,2-diphenyl-1-picrylhydrazyl
ROS	Reactive Oxygen Species
ATP	adenosine triphosphate
MRIZP	Maize Research Institute Zemun Polje
ISTA	International Seed Testing Association
PCA	Principal Component Analysis
ANN	Artificial Neural Network
