# Supplementary material for: Hydropriming-Mediated Metabolic Activation and Vigour Enhancement in Selected Maize (Zea mays L.) Landraces: Application in Ex Situ Conservation
Source: Plants (Basel). 2026 Nov 6;15(21):3479. doi: 10.3390/plants15213479 (PMC13765748; doi:10.3390/plants15213479)
Supplement: Supplementary file 1 [file plants-15-03479-s001.zip › plants-4639873-supplementary.pdf]

plants

MDPI

Supplementary Materials

Table S1. Two-way analysis of variance (ANOVA) *F*-statistics, significance levels, and coefficients of variation (CV) for the evaluated morpho-physiological and biochemical traits in local maize landraces under hydropriming treatments. Legend: G–Genotype; T–Hydropriming treatment; G × T–Genotype-by-Treatment interaction; CV–Coefficient of variation; GC–germination capacity; RL–Root length; SH–Shoot length; RFW–Root fresh weight; SFW–Shoot fresh weight; RDW–Root dry weight; SDW–Shoot dry weight; Pphy–Phytate phosphorus; GSH–Glutathione; Tot Phenol–Total phenolics; YP–Yellow pigment; RSA–Radical scavenging activity; Chl–Chlorophyll. Significance levels according to two-way ANOVA: *–$p \leq 0.05$; **– $p \leq 0.01$; ***– $p \leq 0.001$; ns–not significant ($p > 0.05$). Values in the table represent *F*-statistics.

		***F*-value**			**Pr (>*F*)**			
**Parameter**	**Tissue**	**G**	**T**	**G × T**	**G**	**T**	**G × T**	**CV (%)**
GC	Seed	25.393	1.118	1.339	1.676 × e^{-10} ***	0.3637 ns	0.2004 ns	11.85
RL	Root	5.7007	11.9907	3.0415	0.0018 **	1.301 × e^{-10} ***	0.007 ***	21.31
SL	Shoot	60.4853	1.6899	1.7563	< 2.2 × e^{-16} ***	0.1405 ns	0.0556 ns	16.33
RFW	Root	83.7992	7.8526	2.1604	< 2.2 × e^{-16} ***	3.714 × e^{-06} ***	0,0147 *	21.40
SFW	Shoot	119.0351	4.3877	2.3251	< 2.2 × e^{-16} ***	0.0011 **	0.0084 **	19.75
RDW	Root	106.3816	6.3982	2.2922	< 2.2 × e^{-16} ***	3.565 × e^{-05} ***	0.0094 **	21.11
SDW	Shoot	185.3050	7.8237	2.4973	< 2.2 × e^{-16} ***	3.879 × e^{-06} ***	0.0047 **	17.19
Pphy	Root	310.056	33.970	29.044	< 2.2 × e^{-16} ***	< 2.2 × e^{-16} ***	< 2.2 × e^{-16} ***	7.63
Pphy	Shoot	72.902	601.446	19.969	< 2.2 × e^{-16} ***	< 2.2 × e^{-16} ***	< 2.2 × e^{-16} ***	5.56
GSH	Root	92.860	50.879	33.727	< 2.2 × e^{-16} ***	< 2.2 × e^{-16} ***	< 2.2 × e^{-16} ***	9.20
GSH	Shoot	213.190	10.298	13.618	< 2.2 × e-16 ***	1.158 × e^{-07} ***	2.178 × e^{-14} ***	6.90
Tot Phenol.	Root	141.856	503.104	31.384	< 2.2 × e^{-16} ***	< 2.2 × e^{-16} ***	< 2.2 × e^{-16} ***	4.08
Tot Phenol.	Shoot	135.375	960.182	27.813	< 2.2 × e^{-16} ***	< 2.2 × e^{-16} ***	< 2.2 × e^{-16} ***	3.76
YP	Root	99.312	5592.272	24.758	< 2.2 × e^{-16} ***	< 2.2 × e^{-16} ***	< 2.2 × e^{-16} ***	3.78
YP	Shoot	668.29	14499.55	238.79	< 2.2 × e^{-16} ***	< 2.2 × e^{-16} ***	< 2.2 × e^{-16} ***	1.83
RSA	Root	20.943	20.756	22.702	3.173 × e^{-09} ***	1.244 × e^{-12} ***	< 2.2 × e^{-16} ***	2.85
RSA	Shoot	42.881	212.324	35.276	1.58 × e^{-14} ***	1.244 × e^{-12} ***	< 2.2 × e^{-16} ***	1.82
Chl *a*	Shoot	5057.8	6748.1	1034.3	< 2.2 × e^{-16} ***	< 2.2 × e^{-16} ***	< 2.2 × e^{-16} ***	3.43
Chl *b*	Shoot	524.78	832.64	185.13	< 2.2 × e^{-16} ***	< 2.2 × e^{-16} ***	< 2.2 × e^{-16} ***	6.80
Chl_{tot}	Shoot	2898.41	4058.02	675.12	< 2.2 × e^{-16} ***	< 2.2 × e^{-16} ***	< 2.2 × e^{-16} ***	4.00

Table S2. Contribution of variables (%) to the first two principal components (PC1 and PC2) for: **a**) seedling root and **b**) seedling shoot. Legend: PC1 and PC2 represent the first and second principal components, respectively. Values indicate the percentage of variance contributed by each parameter to the respective component.

	**a**	
**Parameters**	**PC1**	**PC2**
Germination Capacity (GC)	12.76	10.43
Root length (RL)	2.65	19.26
Root fresh weight (RFW)	22.88	5.80
Root dry weight (RDW)	21.47	5.87
Root phytate phosphorus (R_Pphy)	15.41	0.03
Root total glutathione (R_GSH)	0.94	15.82
Root total phenolics (R_TOT PHENOL)	10.77	18.10
Root yellow pigment (R_YP)	11.40	18.02
Root radical scavenging activity (R_RSA)	1.72	6.67
	**b**	
**Parameters**	**PC1**	**PC2**
Germination Capacity (GC)	3.96	13.69
Shoot length (SL)	6.10	12.18
Shoot fresh weight (SFW)	5.70	14.55
Shoot dry weight (SDW)	5.13	14.82
Shoot phytate phosphorus (S_Pphy)	9.80	3.80
Shoot total glutathione (S_GSH)	3.83	14.70
Shoot total phenolics (S_TOT PHENOL)	7.37	9.18
Shoot yellow pigment (S_YP)	8.82	10.53
Shoot radical scavenging activity (S_RSA)	9.88	2.59
Shoot chlorophyll *a* (S_Chl *a*)	13.77	0.95
Shoot chlorophyll *b* (S_Chl *b*)	12.17	1.85
Shoot total chlorophyll (S_Chl tot)	13.47	1.15

Table S3. Artificial Neural Network architecture and data partitioning.

Plant Organ / Model	Predictors	Output Traits	Hidden Neurons	Training *n* (%)	Testing *n* (%)
Root	Genotype identity, Temperature, Immersion duration	9	3	21 (75%)	7 (25%)
Shoot	Genotype identity, Temperature, Immersion duration	12	3	21 (75%)	7 (25%)

Notes: *n* represents the number of experimental mean profiles/observations used per fold in the 4-fold cross-validation scheme ($k = 4$), corresponding to a 75:25 stratified train-to-test dataset partition ($N = 28$ total treatment-genotype combinations per organ system; exactly 21 training and 7 testing observations per fold without rounding residuals). Predictor variables within the network architecture encompass Genotypic Identity, Priming Temperature, and Immersion Duration. Output traits define the total number of evaluated biological endpoints per organ system, while hidden neurons are structurally fixed within a single-hidden-layer Multilayer Perceptron (MLP) framework constrained to exactly three hidden neurons and optimized via an L_2 regularization penalty (weight decay = 0.05).

Table S4. Performance metrics and relative predictor importance (%) of the Multi-Layer Perceptron (MLP) neural network models governing maize root responses under hydropriming regimes.

Target Outputs	R^2	RMSE	MAE	GI (%)	PT (%)	ID (%)
GC	0.907	0.303	0.233	99.00	1.00	0.00
RL	0.770	0.475	0.391	98.56	0.00	1.44
RFW	0.775	0.467	0.398	76.94	0.00	23.06
RDW	0.953	0.217	0.151	51.76	0.00	48.24
R_Pphy	0.906	0.311	0.260	95.40	0.00	4.60
R_GSH	0.792	0.456	0.332	88.00	0.00	12.00
R_TOT_PHENOL	0.963	0.195	0.156	96.20	0.00	3.80
R_YP	0.988	0.116	0.088	97.10	0.00	2.90
R_RSA	0.644	0.588	0.477	89.50	0.00	10.50

Notes: Values R^2 represent trait-specific coefficient of determination; RMSE–root mean square error; MAE–mean absolute error evaluated on Z-score standardise-scaled datasets, where Target Outputs define the comprehensive suite of modeled radicle traits, and relative predictor importance partitioning (%) computed via MLP–multilayer perceptron networks. Abbreviations: GI–genotypic identity; PT–priming temperature; ID–immersion duration; Prefixes R_ denote root tissue parameters; GC–germination capacity; RL–root length; FW–root fresh weight; DW–root dry weight; Pphy–phytate phosphorus; GSH–total glutathione; TOT_PHENOL–total phenolics; YP–yellow pigment; RSA–radical scavenging activity. Data partitioning is executed via a 4-fold cross-validation scheme (k = 4) across a total sample size of N = 28 treatment-genotype nodes (training set n = 21, testing set n = 7 observations per fold).

Table S5. Performance metrics and relative predictor importance (%) of the Multi-Layer Perceptron (MLP) neural network models governing maize shoot responses under hydropriming regimes.

Target Outputs	R^2	RMSE	MAE	GI (%)	PT (%)	ID (%)
GC	0.907	0.303	0.233	99.00	1.00	0.00
SL	0.900	0.312	0.245	96.21	0.00	3.79
SFW	0.916	0.285	0.203	75.18	0.00	24.82
SDW	0.959	0.201	0.153	77.25	0.00	22.75
S_Pphy	0.945	0.234	0.179	100.00	0.00	0.00
S_GSH	0.814	0.425	0.345	40.09	58.92	0.99
S_TOT_PHENOL	0.936	0.251	0.216	98.10	1.90	0.00
S_YP	0.972	0.170	0.136	96.50	3.50	0.00
S_RSA	0.868	0.359	0.314	75.50	24.50	0.00
S_Chl_*a*	0.935	0.255	0.202	36.07	63.93	0.00
S_Chl_*b*	0.924	0.275	0.211	94.67	5.33	0.00
S_Chl_tot	0.961	0.202	0.165	94.80	0.00	5.20

Notes: Values R^2 represent trait-specific coefficient of determination; RMSE–root mean square error; MAE–mean absolute error evaluated on Z-score standardise-scaled datasets, where Target Outputs define the comprehensive suite of modeled shoot traits, and relative predictor importance partitioning (%) computed via MLP–multilayer perceptron networks. Abbreviations: GI–genotypic identity; PT–priming temperature; ID–immersion duration; Prefixes S_ denote shoot tissue parameters; GC–Germination capacity; SL– Shoot length; FW–fresh weight; DW–dry weight; Pphy–phytate phosphorus; GSH–total glutathione; TOT_PHENOL–total phenolics; YP–yellow pigment; RSA–radical scavenging activity; Chl–chlorophyll (*a*, *b*, tot). Data partitioning is executed via a 4-fold cross-validation scheme (k = 4) across a total sample size of N = 28 treatment-genotype nodes (training set n = 21, testing set n = 7 observations per fold).

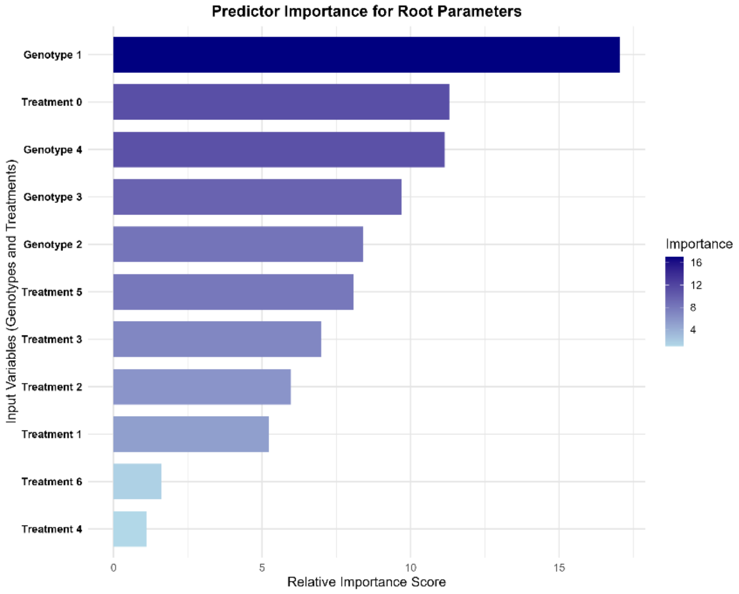

Figure S1. Relative predictor importance for root parameters. Predictor importance scores extracted from the artificial neural network model quantify the non-linear relative predictor importance of landrace identity (Genotypes L1–L4) and hydropriming conditions (Treatments T0–T6) to total variance in root morpho-physiological parameters and metabolic markers. Treatment codes: T0 (unprimed control); T1 (20 °C, 8 h); T2 (20 °C, 16 h); T3 (20 °C, 24 h); T4 (30 °C, 8 h); T5 (30 °C, 16 h); T6 (30 °C, 24 h). Darker bar shading corresponds to higher relative predictor weight.

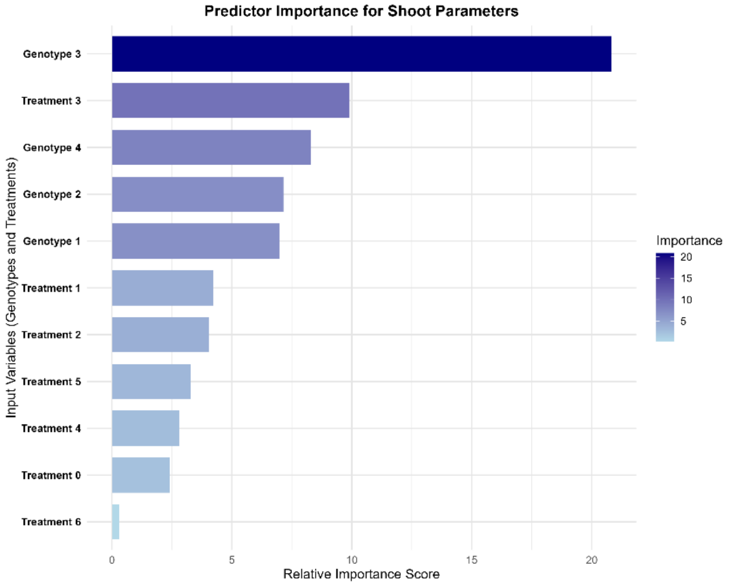

Figure S2. Relative predictor importance for shoot parameters. Predictor importance scores extracted from the artificial neural network model quantify the non-linear relative predictor importance of landrace identity (Genotypes L1–L4) and hydropriming conditions (Treatments T0–T6) to total variance in shoot morpho-physiological parameters and metabolic markers. Treatment codes: T0 (unprimed control); T1 (20 °C, 8 h); T2 (20 °C, 16 h); T3 (20 °C, 24 h); T4 (30 °C, 8 h); T5 (30 °C, 16 h); T6 (30 °C, 24 h). Darker bar shading corresponds to higher relative predictor weight.
